# Increased chromatin accessibility facilitates intron retention in specific cell differentiation states

**DOI:** 10.1093/nar/gkac994

**Published:** 2022-11-10

**Authors:** Veronika Petrova, Renhua Song, DEEP Consortium, Karl J V Nordström, Jörn Walter, Justin J L Wong, Nicola J Armstrong, John E J Rasko, Ulf Schmitz

**Affiliations:** Computational BioMedicine Laboratory Centenary Institute, The University of Sydney, Camperdown 2050, Australia; Gene and Stem Cell Therapy Program Centenary Institute, The University of Sydney, Camperdown 2050, Australia; Epigenetics and RNA Biology Program Centenary Institute, The University of Sydney, Camperdown 2050, Australia; Faculty of Medicine and Health, The University of Sydney, Camperdown 2050, Australia; Laboratory of EpiGenetics, Saarland University, Campus A2 4, D-66123 Saarbrücken, Germany; Laboratory of EpiGenetics, Saarland University, Campus A2 4, D-66123 Saarbrücken, Germany; Epigenetics and RNA Biology Program Centenary Institute, The University of Sydney, Camperdown 2050, Australia; Faculty of Medicine and Health, The University of Sydney, Camperdown 2050, Australia; Mathematics and Statistics, Curtin University, Bentley, WA 6102, Australia; Gene and Stem Cell Therapy Program Centenary Institute, The University of Sydney, Camperdown 2050, Australia; Faculty of Medicine and Health, The University of Sydney, Camperdown 2050, Australia; Cell and Molecular Therapies, Royal Prince Alfred Hospital, Camperdown 2050, Australia; Computational BioMedicine Laboratory Centenary Institute, The University of Sydney, Camperdown 2050, Australia; Department of Molecular and Cell Biology, College of Public Health, Medical and Veterinary Sciences, James Cook University, Townsville, QLD 4811, Australia; Centre for Tropical Bioinformatics and Molecular Biology, Australian Institute of Tropical Health and Medicine, James Cook University, Cairns 4878, Australia

## Abstract

Dynamic intron retention (IR) in vertebrate cells is of widespread biological importance. Aberrant IR is associated with numerous human diseases including several cancers. Despite consistent reports demonstrating that intrinsic sequence features can help introns evade splicing, conflicting findings about cell type- or condition-specific IR regulation by *trans*-regulatory and epigenetic mechanisms demand an unbiased and systematic analysis of IR in a controlled experimental setting. We integrated matched mRNA sequencing (mRNA-Seq), whole-genome bisulfite sequencing (WGBS), nucleosome occupancy methylome sequencing (NOMe-Seq) and chromatin immunoprecipitation sequencing (ChIP-Seq) data from primary human myeloid and lymphoid cells. Using these multi-omics data and machine learning, we trained two complementary models to determine the role of epigenetic factors in the regulation of IR in cells of the innate immune system. We show that increased chromatin accessibility, as revealed by nucleosome-free regions, contributes substantially to the retention of introns in a cell-specific manner. We also confirm that intrinsic characteristics of introns are key for them to evade splicing. This study suggests an important role for chromatin architecture in IR regulation. With an increasing appreciation that pathogenic alterations are linked to RNA processing, our findings may provide useful insights for the development of novel therapeutic approaches that target aberrant splicing.

## Graphical Abstract

**Graphical Abstract:**
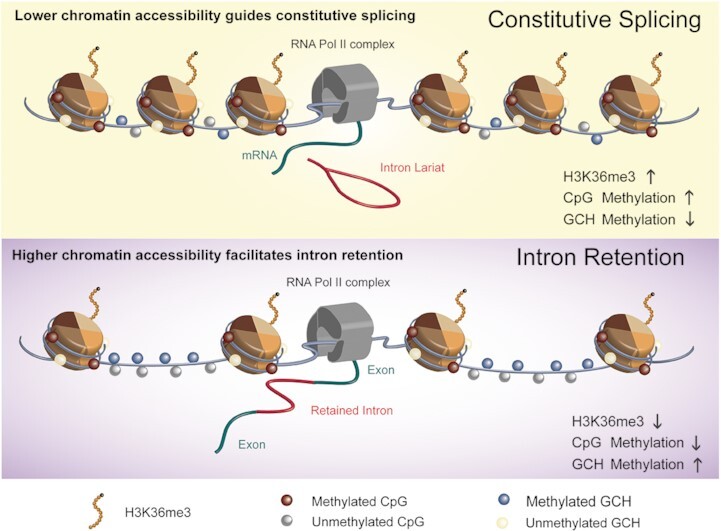
Role of chromatin accessibility in intron retention regulation. Loose positioning of nucleosomes accelerates transcription, facilitating intron retention. Retained introns are also associated with lower CpG methylation and H3K36me3.

## INTRODUCTION

The role of introns in mammalian genomes remains largely unexplained. Given the time and energy required for the transcription and subsequent excision of introns from pre-mRNA, it was important to recognize in recent years that introns can be selectively retained in mature mRNA transcripts and thereby contribute significantly to transcriptomic complexity (1,2). Intron retention (IR) is a form of alternative splicing that was assumed to occur due to the failure of the spliceosome to excise an intron from a pre-mRNA transcript. However, growing evidence suggests that IR is highly regulated by multiple complementary factors (3).

IR is widespread across human tissues and affects >80% of protein-coding genes (4). For example, dynamic IR profiles have been identified in key genes involved in haematopoietic cell differentiation and activation (1,5–8). Fates of intron-retaining transcripts can be diverse and include (i) nonsense-mediated decay triggered by intronic premature termination codons, (ii) detention in the nucleus or nuclear degradation and (iii) translation into alternative protein isoforms or creation of neoepitopes (3,9,10). A better understanding of how IR is regulated is crucial to determining factors leading to aberrant IR, which has been associated with multiple diseases including cancer (11–13).

Despite numerous studies that describe the role of retained introns in key biological functions in animal and human diseases (3,9,11), a comprehensive understanding of their regulation is still lacking. Retained introns have conserved intrinsic characteristics such as a higher GC content, shorter lengths and weaker splice sites in comparison with their non-retained counterparts (2,3,14). These features predispose introns to retention but cannot explain the dynamic IR profiles observed in numerous biological processes. The regulation of alternative splicing has been the focus of many studies. Evidence suggests that alternative splicing is regulated at least at two levels: (i) locally, where *trans*-acting splicing regulators interact with *cis*-acting regulatory elements; and (ii) globally, through the structure of chromatin, which is largely governed by epigenetic factors, including nucleosome assembly, histone modifications and CpG methylation (15). Previous reports have shown that, apart from intrinsic sequence-based features, intron expression can be regulated through RNA-binding proteins (RBPs) and core components of the splicing machinery (1,4), as well as changes to the RNA polymerase II (Pol II) elongation rate (16). Moreover, an increasing number of studies have found links between epigenetic profiles and IR; reporting that IR is associated with reduced CpG methylation (8,17–19) and various histone modifications (20,21). However, these reports have typically established the association of IR with only one epigenetic factor at a time. The question of whether there are dominant epigenetic factors that underpin IR regulation remains unanswered.

In the quest to find a splicing regulatory ‘code’, several studies have used machine learning methods to train models that predict exon usage with increasing precision (22,23). Moreover, some models were developed to predict cryptic splicing events caused by genetic variations and to link these to human diseases (24–26). However, the computational prediction of IR events has not been attempted to date, and the role of epigenetic marks has rarely been considered in computational models of splicing regulation (3,27).

**Figure 1.**
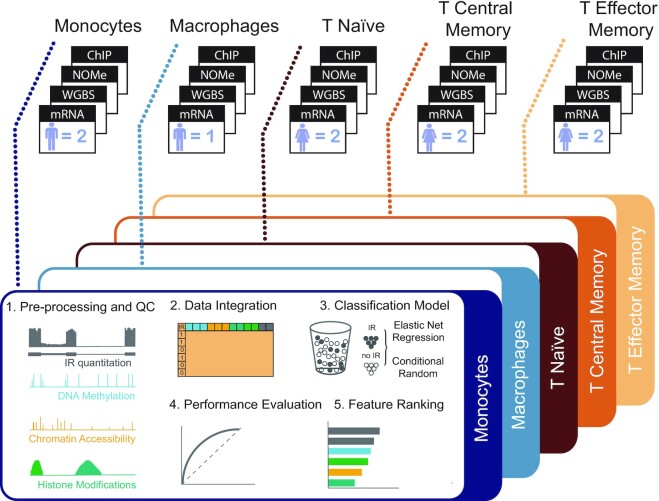
Experimental design and computational workflow to determine regulators of IR. Raw high-throughput data were processed for each biological replicate and amalgamated by cell type from the indicated number of samples (*n*). The output was used for feature extraction: IR events were treated as a binary outcome and we trained an elastic net regression model and a conditional random forest model with a total of 48 sequence-based and epigenetic features. Using feature ranking, we identified the factors that were most strongly associated with IR outcomes and compared the performances of both modelling strategies. These steps were repeated for each cell type.

In this study, we sought to systematically elucidate the role of epigenetic marks in the regulation of IR. We analysed genome-wide profiles of six histone modifications, CpG methylation and nucleosome occupancy at single-base resolution in primary lymphoid and myeloid cells. Using machine learning, we developed models that predict IR in primary human immune cells. More specifically, we trained a logistic regression with an elastic net (EN) classifier and a conditional random forest (cRF) classifier with matched transcriptomic and epigenomic data from monocytes, macrophages, naïve T cells, central memory T cells and effector memory T cells (Figure 1). Our results show that intrinsic characteristics are key for introns to evade splicing and that epigenetic marks modulate IR levels in a cell type-specific manner, where the dominant factor for dynamic IR regulation is chromatin organisation.

## MATERIALS AND METHODS

### Multi-omics data analysis

To investigate how IR is regulated in primary immune cells, we integrated epigenomic and transcriptomic data from the German Epigenome Program (DEEP). Primary monocytes and T cells (naïve, central memory and effector memory) were retrieved from two healthy donors. Monocyte-derived macrophages were retrieved as follows: elutriated monocytes were seeded at 1 × 10^6^ cells/ml in macrophage serum-free medium (Invitrogen) supplemented with 50 ng/ml recombinant human monocyte colony-stimulating factor (rhMCSF; R&D Systems). Cells were incubated at 37°C, 5% CO_2_ for 5 days before macrophage cells were collected. Cell isolation, differentiation, DNA/RNA extraction and library preparation for mRNA sequencing (mRNA-Seq), whole-genome bisulfite sequencing (WGBS), nucleosome occupancy methylome sequencing (NOMe-Seq) and chromatin immunoprecitiation sequencing (ChIP-Seq) experiments are described in detail in these articles (28,29). Donors gave their written and informed consent prior to participating in the study. The study was approved by the ethics committees of the University Hospital Regensburg (Ethikkommission der medizinischen Fakultät, proposal 08/119) and the Charite Universitaetsmedizin Berlin (application numbers EA1/116/13 and EA1/105/09).

#### mRNA-Seq data processing and identification of IR events

RNA-Seq reads (FASTQ format) of each technical replicate were tested for quality using FastQC v.0.11.5 (github.com/s-andrews/FastQC). Further processing, including adaptor trimming, was performed within the IRFinder algorithm for IR quantification (4). Sequencing reads were mapped to the human reference genome (GRCh38, v86) using STAR v2.7 with default parameters (30). IRratios, a quantitative measure of IR levels, were determined as:

$$\begin{equation*}IRratio{\rm{\ }} = {\rm{\ }}\frac{{{\rm{\ }}Intronic{\rm{\ }}Abundance}}{{Intronic{\rm{\ }}Abundance{\rm{\ }} + {\rm{\ }}Exonic{\rm{\ }}Abundance}},\end{equation*}$$

where the intronic abundance is defined as the trimmed mean of the reads that map to an intron, after exclusion of features that overlap the intron and removal of the highest and lowest 30% of values. Exonic abundance is defined as the number of reads that map across an exon–exon junction. Library size normalization (i.e. between-sample normalization) was not required as the ratio between intronic and exonic abundance is determined from within the same transcriptome (4).

IRFinder extracts introns from the ENSEMBL transcript annotations (GTF file, hg38, v86) as the region between two exons in any transcript. Regions covered by a gtf feature within an intron were excluded as they could confound an accurate measurement of IR. Introns that were present in at least 10% of a gene's mature mRNA transcripts (IRratio ≥0.1) with an overall intron depth ≥10 and ≥90% read coverage were considered retained. Retained introns were further filtered for those where the flanking exons had a percent spliced in index (PSI) ≥0.9. Non-retained introns were defined as those with an IRratio ≤0.01 and intron depth <10. Only introns from expressed host genes [fragments per kilobase per million (FPKM) ≥1] with a length <10 000 bp were selected for downstream analyses. For the intron classification system introduced by Braunschweig *et al.* (14), additional filtering criteria were applied:

**Table:** 

Type A	Type B	Type C
• Length <10 000 • 5′ and 3′ exon spliced-in • no known overlapping exon • 5′ and 3′ exon type: constitutive	• Length <10000 • 5′ and 3′ exon spliced-in • *known overlapping exon* • 5′ and 3′ exon type: constitutive	• Length <10 000 • 5′ and 3′ exon spliced-in • no known overlapping exon • *5′ or 3′ exon type: alternative*

In contrast to Braunschweig *et al.*, we kept the 10 kb intron length filter for consistency. Our initial assessment indicated that >10 kb long introns are almost exclusively found in non-retained introns and often present the first intron in a transcript.

We used Cufflinks v2.1.1 (31) to estimate gene abundance in FPKM. Only introns from host genes with FPKM ≥1 were selected for the downstream analyses.

#### WGBS data processing

Raw WGBS FASTQ files were assessed for quality using FastQC v.0.11.5 (github.com/s-andrews/FastQC). Standard Illumina adaptors used for the library preparation were trimmed using cutadapt v.1.10 (32) with a quality cut-off of 20 bp and a minimum read length of 30 bp. Trimmed reads were mapped to the GRCh38 reference genome, duplicate reads removed and methylation calling performed using Bismark v.0.19.0 (33). Only CpG sites with a coverage of >5 reads were retained for further analysis.

#### ChIP-Seq data processing

ChIP-Seq data for six histone modifications (H2K27ac, H3K27me3, H3K36me3, H3K4me1, H3K4me3 and H3K9me3) were aligned to the human GRCh38 reference genome using STAR v2.7 (30). Duplicate reads were removed using Picard v.2.18.4 (broadinstitute.github.io/picard/) and further processed using MACS2 v.2.2.6 (34) to identify histone modification peaks, with default parameters and *q*-value cut-off of 0.01. All histone modifications were processed in the ‘narrow peak’ mode to extract peak summit coordinates. Narrow peaks were used because wide peaks frequently tend to spread across retained and non-retained introns. For visualisation in Integrative Genomics Viewer (IGV; 35), we generated coverage tracks using bamCoverage from deepTools2 (36) with the following parameters –binSize 1 –normalizeUsing BPM –effectiveGenomeSize 2913022398 –extendReads 200. For histone mark (HM) line plots, we subtracted ChIP-Seq input from respective HM ChIP-Seq read counts and normalised based on bins per million (BPM) mapped reads using bamCompare and parameters –binSize 1 –scaleFactorsMethod readCount –effectiveGenomeSize 2913022398 –operation subtract –normalizeUsing BPM.

#### NOMe-Seq data processing

Raw FASTQ files were assessed for quality using FastQC v.0.11.5 (github.com/s-andrews/FastQC). Reads were mapped to the GRCh38 reference genome, duplicate reads removed and methylation calling performed using Bismark v.0.19.0 (33). GCH methylation information was extracted with the coverage2cytosine utility with –nome parameter.

Nucleosome-free regions (NFRs) were predicted using the gNOMePeaks tool (37) with default parameters, which include 4000 bp up- and downstream from each peak for background signal calculation and the maximum distance between GpC sites of 150 bp. We used the same algorithms to predict nucleosome positioning by substituting GCH methylation, as required input, with GCH occupancy (1 – GCH methylation) and reducing the background region to 1000 bp up- and downstream from each peak and the distance between GCH sites to 20 bp.

### Elastic net and conditional random forest modelling

#### Feature selection

Model features were associated with three genomic regions around retained and non-retained introns: (i) ±100 bp from the 5′ splice site; (ii) ±100 bp from the 3′ splice site; and (iii) ±100 bp from the middle of an intron, each region being 200 bp long. GC content was extracted using bedtools v.2.26.0 (38) nuc command. For splice site strength calculations, we used MaxEntScan (39). CpG density values were obtained using Repitools (40). The PSI of flanking exons was calculated as described in (41). Exons with PSI ≥0.9 were considered as included. Branch point strength and distance were computed using the SVM-BPfinder algorithm (42).

We considered RBPs as putative *trans*-regulators of dynamic IR. We extracted RBP-binding motif data from the ATtRACT database (attract.cnic.es) (43) and identified motifs of differentially expressed RBPs (myeloid versus lymphoid) that reside within or adjacent to dynamic introns (±100 bp from the 5′ splice site, 3′ splice site or middle of the intron).

To generate epigenetic features, we overlapped three regions of interest with the pre-processed epigenetic data. NFRs were defined as regions >40 bp in length with a *P*-value ≤0.05 (Fisher test comparing CpG methylation in the NFR with the surrounding background). Presence or absence of an NFR was dichotomized as ‘yes’ = 1 and ‘no’ = 0. Information about nucleosome location was included in the model in a similar manner (nucleosomes were defined as regions >140 bp in length with a *P*-value ≤0.05).

The relationship between histone modification and IR was included in the model through the presence or absence of an overlap with a histone signal region. It was categorised as 0 = no overlap, 1 = overlap with a region of HM signal, 2 = overlap with a region of strong signal [strong signal = mean (HM pile-up) + SD (HM pile-up)]. The full list of features is presented in Supplementary Table S1.

#### Model training and validation

To identify features important for IR, we constructed a binary classification model using the EN algorithm. We approached the problem in a naïve manner, i.e. we did not impose any prior assumptions about the factors that might potentially play a role, and therefore an equal penalty factor was applied to all features. EN classification was performed in the caret R package (44) using the glmnet method (45) for a binary outcome. The group imbalance, due to the different number of retained and non-retained introns identified as suitable for modelling, was handled by down-sampling, using the downSample command. Parameter λ, determining the overall size of the regularization penalty, was optimized by the 10-fold cross-validation procedure. Features were ranked based on the absolute values of the model coefficients.

We repeated this *in silico* analysis to validate our results using an independent machine learning algorithm, cRF. In cRF, unlike standard RF where the first split variable is randomly selected, an association test between the outcome and the model predictors is performed first. The ranked *P*-values are then used to identify the covariate with the strongest association with the outcome, which is later used for the first binary split at cutpoint *c* for a continuous covariate or at category *C* for a categorical covariate. cRF classification was also performed in caret using the cforest method as implemented in the party R package (46). The cRF model provides an unbiased measure of variable importance, which we used to rank the most important features for IR prediction.

To avoid overfitting, we ranked the features’ importance using both EN and cRF techniques (47). Moreover, our findings were validated across different blood cell lineages from different humans.

### Analysis of lineage-determining transcription factors

Transcription factor (TF) affinity scores were calculated around the splicing sites of retained and non-retained introns using TEPIC Version 2.2 (48). We also provided information about the open chromatin regions previously predicted by the gNOMePeaks pipeline using NOMe-seq data to the algorithm. We filtered TFs with affinity scores >0.1 and reported those with the highest affinity scores in at least one of the five cell types.

### Statistical analysis

All statistical analyses were performed in R v.4.0. For the identification of differentially retained introns, we used the Audic and Claverie test (49). We have used the IRFinder's built-in Bayesian statistic adapted for digital counts, i.e. the Audic and Claverie test, because of the small number of biological replicates (two for monocyte and T cells, and one for macrophages). *P*-values ≤ 0.05 were considered significant. Clustering was performed using unsupervised hierarchical clustering with complete linkage. Gene Ontology (GO) enrichment analysis on host genes of dynamic introns was performed using the R Bioconductor package enrichR (50).

### Differentially retained introns versus dynamic introns

Dynamic introns are retained in one or more cell types based on our criteria for retention (IRratio ≥0.1; intron depth ≥10; ≥90% read coverage) and not retained in one or more cell types (IRratio ≤0.01; intron depth <10). Differentially retained introns are determined based on pairwise comparisons (cell type A versus cell type B) with Audic and Claverie tests. They need to fulfil the retention criteria in at least one of the two cell types. Introns with a *P*-value ≤0.05 and ΔIR ≥0.1 are considered significantly differentially retained.

## RESULTS

### Intrinsic features of retained introns are consistent across cell types

To investigate how IR is regulated in primary immune cells (CD4+ T cells, monocytes and macrophages), we integrated transcriptomics (mRNA-Seq) data with epigenomics data including genome-wide CpG methylation (WGBS), histone modifications (ChIP-Seq) and nucleosome occupancy (NOMe-Seq) (Supplementary Table S2). The cells were isolated from peripheral blood of two healthy donors, except for the monocyte-derived macrophages. Using the IR identification software IRFinder (4), we quantified IR events of expressed genes (FPKM >1) in five cell types across myeloid and lymphoid cells, representing two modes of differentiation: monocyte to macrophage differentiation and naïve T-cell differentiation into central memory (CM) and effector memory (EM) T cells.

We identified a total of 26 147 retained introns in 12 379 genes, some of which were retained in both myeloid and lymphoid cells while others were cell type specific (Supplementary Figure S1A). The number of retained introns detected was independent of sequencing depth (Supplementary Figure S1B). Consistent with previous reports, retained introns in our dataset are shorter in length, exhibit a higher GC content and have weaker splice site strengths compared with non-retained introns (Supplementary Figure S1C). In addition, we found that retained introns have weaker branch points that are on average further away from the adjacent splice site compared with branch points in non-retained introns (Supplementary Figure S1D).

**Figure 2.**
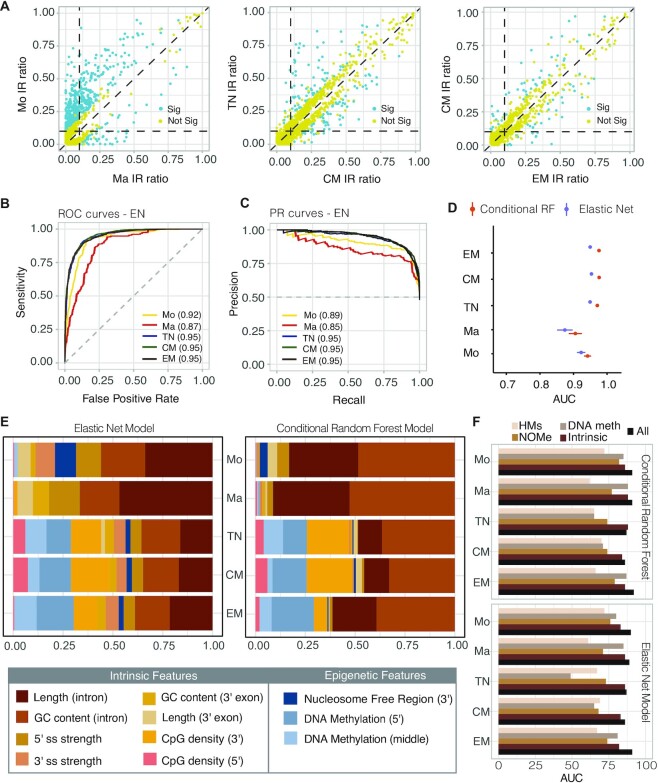
IR prediction and model feature association analyses. (**A**) Scatter plots of differential IR events (blue, significant; yellow, not significant) between monocytes (Mo) versus macrophages (Ma) (left), naïve (TN) versus central memory (CM) T cells (middle), and CM versus effector memory (EM) T cells (right). The scatter plots include only those introns with an IR difference of ΔIR ≥0.1 and an IRratio >0.1 in one of the two cell types. (**B**) Receiver operating characteristic (ROC) curves and (**C**) precision recall (PR) curves comparing the performance of the EN classifier in five cell types. (**D**) Comparison of AUC values between EN and cRF algorithms; error bars show the 95% confidence interval. (**E**) Variable importance scores for the top 10 features identified by EN and cRF algorithms. The scores were scaled to values that add up to 1.0 and the size of a bar corresponds to the effect size. (**F**) Model performance using feature subsets (HM, histone modification; DNA meth, DNA methylation).

Our analysis revealed diverse splicing patterns in myeloid and lymphoid cells. While 40% of the retained introns in myeloid cells were significantly differentially retained (ΔIR ≥0.1; *P* <0.05 Audic–Claverie test) between monocytes and macrophages (571/1425), T cells displayed greater stability regarding IR, with only 8% of introns classified as differentially retained (146/1812 in naïve T versus CM, and 80/969 in CM versus EM). In contrast to the monocyte to macrophage differentiation, where we observed a reduction in IR events (Figure 2A), the overall number of retained introns remained consistent in all CD4+ T cells. These patterns coincide with fewer differences in gene expression amongst T-cell types in contrast to major gene expression changes in monocyte to macrophage differentiation (Supplementary Figure S1E). Interestingly, more dynamic IR profiles have previously been described in the context of CD4+ T-cell activation which coincided with marked differences in gene expression (6). Supplementary Figure S1F shows expression profiles of the genes harbouring introns that remain non-retained during monocyte to macrophage differentiation or T-cell maturation.

Most retained introns in our analysis overlapped with HMs, i.e. H3K9me3, H3K27me3, H3K27ac, H3K36me3, H3K4me1 and H3K4me3, or with an NFR (predicted from NOMe-seq data) located around their 5′ and 3′ splice sites (±100 bp) as well as the middle of an intron (Supplementary Figure S2A). Interestingly, many non-retained introns (∼50%) lacked such epigenetic marks in lymphoid cells (as opposed to only 20–30% of retained introns). H3K36me3 was the most frequently observed histone modification followed by NFR peaks. In retained introns, between 30% and 60% of H3K36me3 signals were classified as strong (see the Materials and Methods), whilst in non-retained introns, the proportion of overlap with the regions of strong signal ranged between 2% and 18% (Supplementary Table S3).

CpG methylation profiles (extracted from WGBS data) for retained and non-retained introns displayed a characteristic bimodal distribution, with two distinct peaks at 0% and 100%. Differential methylation was predominantly found at the splice sites when we compared regions of genomic DNA associated with IR and no IR. At the 5′ splice sites, we observed higher methylation levels in non-retained compared with retained introns in all five cell types. However, there was a cell type specificity in terms of CpG methylation within introns and 3′ splice sites (Supplementary Figure S2B).

The M.CviPI enzyme, used in NOMe-seq experiments, methylates GpC sites that are not bound by nucleosomes. GCH methylation levels (where H is any nucleobase except guanine) provide information about chromatin accessibility. Unlike endogenous CpG methylation, GC dinucleotides are rarely fully methylated (51), therefore mid-range levels (>20%) are usually sufficient to indicate open chromatin regions. In our data, chromatin accessibility (i.e. GCH methylation) increased from monocytes to macrophages, with slightly higher levels in non-retained introns, while lymphoid cells had increased chromatin accessibility (GCH methylation levels 15–35%) but with higher levels in retained introns compared with non-retained introns (Supplementary Figure S2C).

To determine important factors for IR regulation, we compiled a list of features grouped into different classes: (i) sequence-based features: intron length, GC content, splice site strength, CpG density, branch point strength and distance (also referred to as intrinsic features); (ii) transcriptomics features: PSI values of the flanking exons; and (iii) epigenomics features extracted from the WGBS, ChIP-Seq (H3K9me3, H3K27me3, H3K27ac, H3K36me3, H3K4me1 and H3K4me3) and NOMe-Seq data (Supplementary Table S1). We then used these features (*n* = 48) to train EN models for each cell type and predict whether introns are either retained or non-retained. The performance of our models was assessed based on the area under the receiver operating characteristic curve (AUC) values, which ranged between 0.87 and 0.95 (Figure 2B) and values for the area under the precision recall (PR) curve (accuracy) ranging between 0.85 and 0.95 (Figure 2C). The consistently high values suggest that the model choice was appropriate for the task.

The EN model assumes a monotonic linear relationship between the class variable and the model features. To determine whether this assumption is adequate for IR classification, we also trained cRF models, which do not make any prior assumption about the relationship between the outcome of interest and the model features. Comparing the results from both types of models, we found that cRF performed slightly better than EN, with AUC values ranging between 0.91 and 0.98 (Figure 2D; Supplementary Figure S3A) and PR values between 0.87 and 0.95 (Supplementary Figure S3B).

Next, to evaluate whether the learned relationship between the model features and IR was generalisable across cell types, we trained our model with data from one cell type and tested it with data from another cell type. For all training/test data pairs, the AUC and accuracy metrics were comparable with those models that were trained and tested on the same cell type (Supplementary Table S4).

To assess which features contribute most to the model performance (and thus the relevance of a feature to IR), we used variable-importance measures (VIMs). For EN, these are the regression coefficients ordered from lowest to highest, where parameters with larger values have a greater effect. For cRF, variable importance was calculated as the mean decrease in accuracy after permutation of each model feature. Given the known properties of retained introns, it was no surprise that intrinsic features, such as length, GC content and CpG density, were ranked as the top predictors, with a high level of agreement across all cell types analysed (Figure 2E). Again, we observed consistency between the EN and cRF models, except for minor variations in the order in which important features were ranked.

Epigenetic features were also ranked among the top five predictors across all models and cell types; however, their nature and relative importance varied between cell types (Figure 2E). Overall, EN models ranked epigenetic features as moderately to very important (VIM between 0.4 and 0.8), which is comparable with the intrinsic features (ranging between 0.3 and 1). In contrast, cRF identified epigenetic features as somewhat important, with VIM mostly below 0.50 (Supplementary Figure S3C, D). Nevertheless, intrinsic features were consistently identified as most relevant for correctly classifying IR, suggesting that these features predispose introns to be retained irrespective of cell or tissue type. Therefore, it was no surprise that model performances dropped only slightly when trained with intrinsic features only and poorer performances were observed in models trained with epigenetic features only (Figure 2F).

We also investigated whether different types of introns may have evolved different forms of regulation, with different features involved. Braunschweig *et al.* proposed three types of introns (Type A, B and C) that differ not only in their intrinsic sequence-based features but also in their relative levels of inclusion and in the impact their inclusion has on resulting transcripts (14). The majority, i.e. 96%, of the introns included in our analyses represent Type A introns. These introns are flanked by constitutive exons. Type A introns that are retained have a higher GC content and shorter length compared with non-retained introns in that class, thus following the same trend as observed for all retained introns (Supplementary Figure S4A). Type B introns overlap with annotated exons from other isoforms or an antisense gene. Differences in length between retained and non-retained Type B introns are smaller than in Type A introns and insignificant in macrophages, T-naïve and T-effector memory cells. Likewise, the differences in GC content are also smaller and insignificant in macrophages. Intrinsic features of Type C introns, which are flanked by alternative exons, resemble those of Type A introns (Supplementary Figure S4A). The sample size of non-retained Type B and retained Type C introns were too small for model-based classification (Supplementary Table S5). Therefore, feature importance could only be determined for Type A introns, which returned the results concordant to those described above.

To further check if our results might be biased by a certain dominant group of introns, we returned to the original set of retained and non-retained introns and divided it into bins - first, by intron length and then, by the host gene expression (Supplementary Figure S4B, C). We then performed machine learning on each of those bins: short introns (<100 nt), medium introns (100–500 nt) and long introns (>500 nt), as well as genes expressed at a low (1 ≤FPKM <25), medium (25 ≤FPKM <75) and high level (FPKM ≥75). Variable importance analysis revealed very similar results for all introns, with intrinsic features (i.e. GC content and length) consistently topping the list of IR predictors (Supplementary Table S6). These results support the idea that intrinsic features predispose introns to retention, irrespective of their length or transcriptional activity of the host gene.

Though, when we compared intron characteristics between the different classes we found significant differences in GC content and length, except for Type A and B introns, which have similar GC and length profiles (Supplementary Figure S5).

### Chromatin accessibility is predicted to be the strongest regulator of IR

In the previous section, we classified IR on a cell type-specific basis and determined the intrinsic features as having the strongest association with IR outcomes. However, we often find that an intron is retained in one cell type but not in another. In those cases, factors beyond intrinsic features are the likely drivers of this transition.

**Figure 3.**
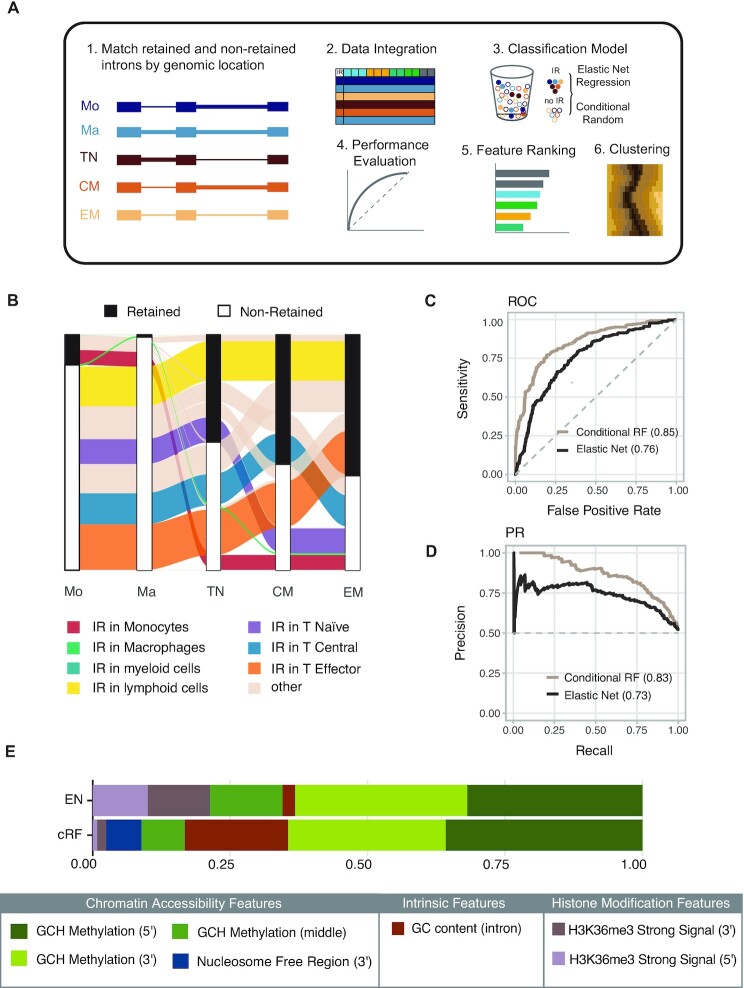
Analysis of dynamic intron retention. (**A**) Modified modelling strategy from Figure 1. Only introns that were found to be in retained and non-retained states in different cell types were included in the analysis. (**B**) Alluvial plot illustrating the dynamics of IR states among the five cell types (*n* = 1540). (**C**) ROC and (**D**) PR curves comparing the performance of cRF (brown) and EN (black) trained with features associated with dynamic introns. (**E**) Variable importance scores for the top five features identified by EN and cRF algorithms, scaled between 0 and 1.

To find these IR determinants, we modified our initial modelling approach by focusing only on the dynamic introns—those that changed their retention status between cell types (Figure 3A). In total, 1540 introns matched this criterion with various IR patterns (Figure 3B; Supplementary Figure S6). Results of a GO enrichment analysis suggest that genes hosting dynamic introns are involved in chromosome organisation and RNA processing (Supplementary Figure S7A). Genes with dynamic introns that are differentially expressed between myeloid and lymphoid cells, i.e. those that are putatively affected by changing IR profiles, are also associated with processes including chromosome organisation and RNA processing (Supplementary Figure S7B).

We used dynamic introns to train EN and cRF models with both epigenetic and intrinsic features. The cRF model performed better than the EN model, achieving AUCs of 0.85 and 0.76, respectively (Figure 3C). cRF also achieved a higher area under the PR curve value (0.83) than EN (0.73) (Figure 3D). The poorer performance of EN might be a reflection of the model's inability to fully utilise complex structures within the omics data, thus supporting the notion that a relationship between chromatin modifiers and IR is indeed non-linear, as previously suggested (52). To verify this hypothesis, we used another classifier (SVM, support vector machine) that is able to pick up non-linear relationships as well. Although inferior to the cRF classifier, SVM performed significantly better than the EN classifier, supporting the concept of a non-linear relationship between chromatin modifiers and IR (Supplementary Figure S8).

Evaluation of feature rankings revealed that, despite varying model performances, both EN and cRF models identified features related to chromatin accessibility as most important for correct IR classification (Figure 3E). These features include GCH methylation and GCH (i.e. nucleosome) occupancy, and the presence of NFRs. GCH methylations at the 5′ and 3′ splice sites were determined as the most important features discriminating retained from non-retained introns in both models. Supplementary Table S7 shows the average percentage GCH methylation values at the 5′ and 3′ splice sites of all retained introns and dynamically retained introns. The cRF classifier also identified CpG methylation as somewhat important for IR classification, which has a known relationship with chromatin accessibility (53–55). Interestingly, the cRF model also identified GC content as a moderately important contributor to IR outcomes, whilst the EN model included HMs (H3K27ac and H3K36me3) among the top 10 predictors (Supplementary Figure S9A).

**Figure 4.**
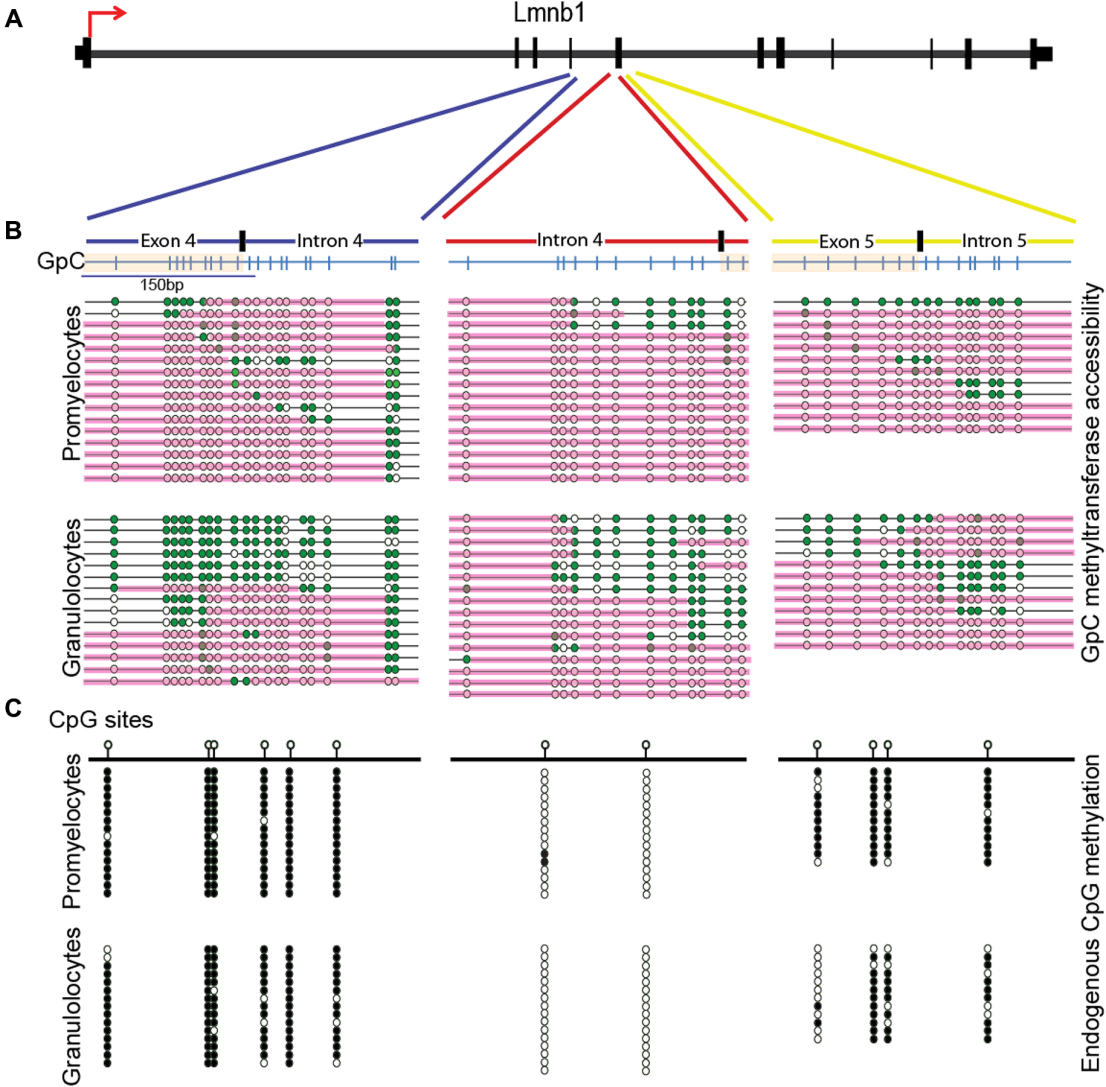
NOMe-Seq analysis of *Lmnb1* exon 4/intron 5. (**A**) Map of *Lmnb1* indicating exons as vertical black lines and introns in between them. The red arrow indicates the direction of transcription. (**B**) Nucleosome occupancy as assessed using GpC methyltransferase (M.CviPI) accessibility. GpC maps of regions spanning exon 4/intron 5 are shown with GpCs indicated by blue vertical lines. Exons are shaded in light orange. Each horizontal line below the maps represents a single allele, with green circles indicating accessible GpCs. Inaccessible GpCs are in white. Pink lines indicate contiguous M.CviPI-inaccessible regions occupied by nucleosomes. There is a 40% depletion of nucleosome occupancy in exon 4/intron 5 in granulocytes compared with promyelocytes. (**C**) Methylation of CpG sites (lollipops), with black circles indicating methylated dinucleotides and white circles indicating unmethylated dinucleotides. A lack of CpG methylation was observed near the exon 5/intron 5 boundary in granulocytes compared with promyelocytes, but not in other regions.

To confirm the importance of chromatin accessibility in IR regulation, we performed gene-specific NOMe-Seq to compare the patterns of DNA methylation and nucleosome occupancy spanning the exonic and intronic regions (exon 4 to intron 7) of *Lmnb1* in promyelocytes and granulocytes. *Lmnb1* (Lamin B1) is known to contain dynamic introns starting from intron 5 and is important during granulopoiesis in mice (1). Our data indicate loss of DNA methylation and nucleosome occupancy in granulocytes, upstream of intron 5, which is where we start to see IR increased in granulocytes (Figure 4). These epigenetic marks are present at higher levels in myeloid progenitors.

Finally, we tested whether RBPs, as putative *trans*-regulators of dynamic IR, can improve our model performance. We extracted RBP-binding motifs from the ATtRACT database (43) and identified motifs that reside within or adjacent to dynamic introns (myeloid versus lymphoid). Only three of these RBPs were differentially expressed between myeloid and lymphoid cells (ENOX1, IGF2BP3 and SAMD4A; *P*-adj. <0.05; log_2_FC >2). We incorporated this information as features in our classification models. The results show that RBP-related features improved the model performance marginally from AUC = 0.85 without RPB features to AUC = 0.882 (with RPB features). The best ranked RBP-related feature was for *IGF2BP3* with binding sites at the centre of dynamic introns (rank #27).

### Epigenetic IR regulation is independent of gene expression regulation

**Figure 5.**
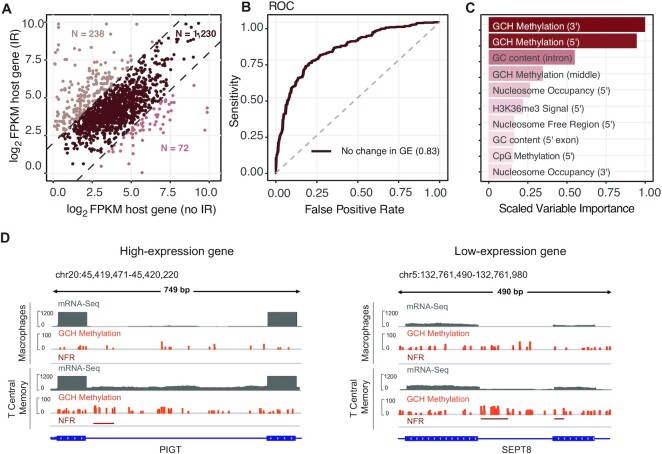
Analysis of introns from genes with non-differential expression levels. (**A**) Scatter plot of host gene expression for introns that change their IR status. Light-coloured dots, separated by dashed diagonal lines, represent differentially expressed genes (FC ≥3; *P* ≤0.05). (**B**) ROC curve indicating the performance of a cRF model fitted on the data from non-differentially expressed genes (GE, gene expression). (**C**) Ranking of the features based on scaled variable importance scores. (**D**) Integrative Genomics Viewer (IGV) plots revealing higher density and hypermethylation levels of GCH sites in the splice site regions of differentially retained introns in both high and low expressed gene examples (NFR, nucleosome-free region; GCH, methylation methylation levels of GC dinucleotides followed by any nucleobase except guanine).

It is reasonable to assume that changes in the epigenetic landscape might not directly affect IR but rather gene expression (56). To confirm that the features identified as relevant to IR are independent from gene expression regulation, we split dynamically retained introns into three groups: (i) host gene expression is reduced along with the change in IR status; (ii) host gene expression remained stable (log_2_FC FPKM ≤2); and (iii) host gene expression increased (Figure 5A). For most of the dynamic introns, we observed only marginal differences in host gene expression (*n* = 1220), whilst down- and up-regulated host genes were associated with 73 and 247 dynamically retained introns, respectively. Correlation analyses suggested that IR ratios of dynamic introns are on average slightly positively correlated with host gene expression (Supplementary Figure S10). We repeated the classification analysis on the group of introns where the IR changes were not accompanied by host gene expression changes. Since the relationship between IR and epigenetic model features is not linear, as was established in the previous section, we only used the cRF algorithm.

The model fitted to this data subset achieved an AUC of 0.83 (Figure 5B) and an area under the PR curve value of 0.78 (Supplementary Figure S9B). The features that were selected as important were GCH methylation at the 5′ and 3′ splice sites and GC content in the same order as in the model trained on all dynamically retained introns (Figure 5C). This observation held true for host genes that were expressed at both a high and a low level (Supplementary Figure S9C). We therefore concluded that the observed epigenetic changes associated with IR modulation are independent from gene expression regulation. In Figure 5D, we show two exemplary introns where greater chromatin accessibility was associated with an increase in IR: phosphatidylinositol glycan anchor biosynthesis class T (PIGT) helps build the glycosylphosphatidylinositol anchor which is found on the surface of various blood cells (Figure 5D, left). *PIGT* is known to express many isoforms through alternative splicing including IR. The nucleotide-binding protein SEPTIN8 is a regulator of cytoskeletal organisation, which has multiple alternatively spliced transcript variants as well (Figure 5D, right).

Next, we assessed whether lineage-determining TFs (LDTFs) might regulate chromatin accessibility in dynamic introns. We analysed TF binding affinities in retained and non-retained introns using TEPIC (48). In total, we identified 21 TFs with differential affinity scores in dynamic introns (Supplementary Table S8). These results suggest that there are multiple instances where the binding affinity of an LDTF is different between retained and non-retained introns. However, it remains to be determined whether these TFs cause changes in chromatin accessibility or whether chromatin accessibility facilitates changes in TF binding affinity.

### Changes in chromatin structure are associated with cell type-specific IR

**Figure 6.**
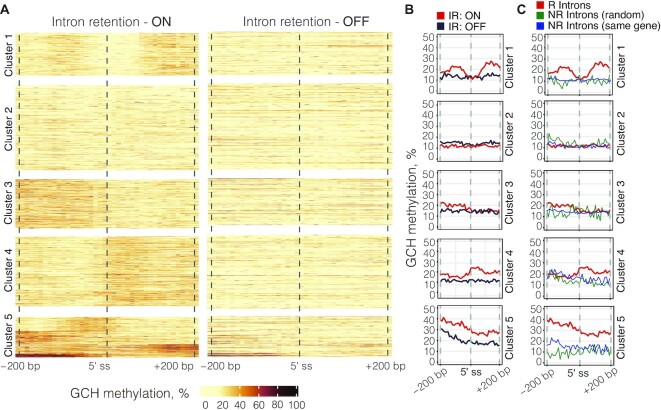
GCH methylation clustering in differentially retained introns. (**A**) Clustering of GCH methylation in the ±200 bp region around the 5′ splice site (ss). Each line corresponds to one intron that is in either a retained (left) or a non-retained state (right). Cluster 1 (*n* = 185), cluster 2 (*n* = 367), cluster 3 (*n* = 211), cluster 4 (*n* = 302), cluster 5 (*n* = 174). (**B**) Line plots showing average GCH methylation values (i.e. chromatin accessibility) in retained versus non-retained introns across five clusters. (**C**) Line plots showing average GCH methylation values in retained versus (random) non-retained introns.

As chromatin accessibility was identified as the strongest predictive factor for dynamic IR, we closely examined its relationship with retained and non-retained introns. We identified five distinct GCH methylation profiles in the ±200 bp region around the 5′ splice site of retained introns (Figure 6A, left). Similar clustering profiles were identified in the region around 3′ splice sites and the middle of introns (Supplementary Figure S11). To understand changes in chromatin status in the context of dynamic IR, we plotted the GCH methylation values of the same introns when they were not retained (Figure 6A). The associated heatmap shows that GCH methylation is widely depleted in non-retained introns, with no distinct clustering. In retained introns, however, we observed a clear increase in GCH methylation immediately upstream or downstream from the 5′ splice site (Figure 6B, clusters 1, 3 and 4). We also identified a group of retained introns with relatively low levels of GCH methylation (cluster 2) and another with particularly strong GCH methylation (cluster 5). To ensure that the observed differences in chromatin accessibility levels are associated with IR, we repeated the analysis and compared GCH methylation levels between dynamic retained introns and i) random non-retained introns across genome, matched by length and GC content; ii) non-retained introns from the same host gene (Figure 6C). The results largely support the trends identified in Figure 6B. Additionally, we explored whether the identified GCH methylation patterns are associated with differences in intrinsic features between introns that comprise those groups (Figure S12A, B & C) or whether the clusters belong to a particular cell type (Figure S12D). Of note, dynamic introns in cluster 1 representative of instances with elevated GCH methylation levels on both sides of the 5′ splice site are characterised by more ‘normal’ intron features, i.e. they are longer and have lower GC and a stronger 5′ splice site than other dynamically retained introns.

**Figure 7.**
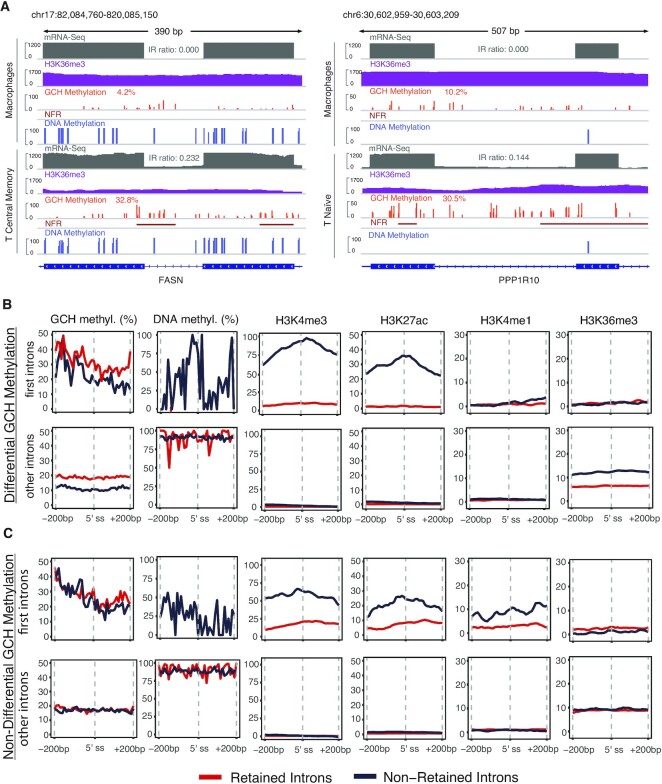
Interplay between chromatin accessibility, CpG methylation and histone modifications. (**A**) IGV plots of mRNA-Seq, H3K36me3 ChIP-Seq, NOMe-Seq and WGBS-Seq data indicate different levels of GCH methylation between retained and non-retained introns and a higher prevalence of NFRs in the regions proximal to IR. (**B**) Line graphs show the average levels of GCH methylation, CpG methylation and the difference between ChIP-Seq H3K4me3, H3K27ac, H3K4me1 and H3K36me3 signals and ChIP-Seq Input, normalised to the BPM, in retained (red) and non-retained (blue) introns associated with chromatin status. The first row shows epigenetic signals at the 5′ splice site of first introns (close to the promoter region) and the second row represents all other introns. (**C**) The same analysis performed in (B) is repeated for introns where the chromatin status remains the same, i.e. non-differential GCH methylation.

Upon visualising the intronic regions that changed their IR status between cell types, we observed greater chromatin accessibility levels in retained introns (Figure 7A). Moreover, for the majority of introns, we found that IR gain was accompanied by a reduction in H3K36me3 signal (Figure 7A). Based on the observed patterns, we hypothesise that there is an association between chromatin dynamics and IR: chromatin is more likely to be in a permissive state (high GCH methylation) in the vicinity of retained introns and more compact (low GCH methylation) around constitutively spliced introns. Indeed, we observed that chromatin becomes more accessible as introns become retained (65% of observations). In other cases, the IR status changes without any change to the chromatin state (35% of observations).

Based on the observations concerning chromatin accessibility, we sought to assess the relationship between IR and epigenetic factors in the context of changing chromatin states, i.e. differential GCH methylation (Figure 7B), and stable chromatin status, i.e. non-differential GCH methylation (Figure 7C). In our analysis, we separated first introns from other introns to detach epigenetic signals associated with gene promoters. The patterns of CpG methylation, and H3K27ac, H3K4me3 and H3K4me1 levels in retained and non-retained introns were similar in both chromatin modes (dynamic and stable). First non-retained introns displayed enrichment for HMs and reduced CpG methylation levels, while first retained introns had negligible levels of HMs and were marked by the absence of CpG methylation (Figure 7B, C, top rows). In contrast, the above-mentioned HMs were silenced in the internal introns irrespective of the IR status, while the H3K36me3 signal increased. Interestingly, H3K36me3 levels were reduced in retained introns associated with dynamic chromatin (Figure 7B, second row, far right), while they remained similar in retained and non-retained introns associated with stable chromatin (Figure 7C, second row, far right). To determine whether H3K36me3 and chromatin accessibility (%GCH) are interdependent, we normalised the H3K3me3 signal by fitting a generalised linear model where H3K3me3 counts depend on GCH methylation values. After removing the nucleosome occupancy effect, we found the H3K3me3 diminished with no differences between retained and non-retained introns, thus confirming that H3K36me3 is interlinked with nucleosome occupancy (Supplementary Figure S13).

One of the most interesting results of this analysis was that there are no differences in epigenetic marks between internal retained and non-retained introns when a stable chromatin state is maintained (Figure 7C, bottom row). This is probably due to other factors, such as RBPs, that modulate IR independent of chromatin accessibility. We also cannot exclude the possibility that there are other as yet undiscovered mechanisms of IR modulation, such as RNA modifications (57), demanding further investigation.

## DISCUSSION

In this study, we have employed a machine learning approach to determine regulators of IR in primary haematopoietic cells. For the first time, we provide integrated matched transcriptomic, nucleosome occupancy, CpG methylation and six histone modification profiles from five primary human cell types representing two independent systems of haematopoietic cell differentiation. Previous studies have described features that are associated with retained introns, including a higher intronic GC content, shorter intron lengths, weaker 5′ and 3′ splice site strengths and some epigenetic marks (2,14,17). Braunschweig *et al.* had previously assessed the importance of intrinsic features (length and GC content) of introns and neighbouring exons, as well as splice site strengths, for IR decisions. Using Kolmogorov–Smirnov statistics and a logistic regression model, they found that *cis*-acting features are predictive of IR in human neural tissues. However, this and other studies have used single or paired omics layers (mRNA-Seq; WGBS) only, missing out on important epigenetic factors. Moreover, they often used individual cell lines for their analyses, missing out on cell type-specific differences in epigenetic IR regulation.

### Machine learning helps determine regulators of alternative splicing

We applied supervised machine learning using EN and cRF algorithms as well as a support vector machine. Unlike deep learning methods, which are very capable of identifying complex relationships but do not provide tools to determine how exactly an outcome was determined (58), these multivariate models allow the identification of features that contribute most to the outcome of interest (IR). Such a modelling strategy is ‘data independent’ and can be applied to other forms of alternative splicing as well. For example, RF has been used to study the importance of chromatin modifications in the interaction between topologically associated domains (59), and EN was used to model prognostic alternative splicing signatures in breast cancer (60).

### Intrinsic features cannot explain dynamic intron retention

Intrinsic features, such as length and GC content, have been consistently reported in association with IR across cell types (1,4) and even across vertebrate species (2,14). Indeed, our models demonstrated that intrinsic features are the dominant predictors of IR even when we trained them with data from one cell type and tested their performance on another cell type. However, since intrinsic features cannot account for dynamic IR changes, we suspected that epigenetic factors modulate IR, which was confirmed by our models when we trained them with features associated with dynamic IR events. When we trained these models with only intrinsic features, the prediction accuracies became rather poor.

However, there are other known factors that can influence the retention of an individual or small groups of introns. These include RBPs (4), RNA Pol II elongation rate (3,17) and decoy exons (i.e. cryptic exons) (61). A recent study by Parra *et al.* has shown that decoy exons interact with splice acceptor sites and thereby block intron excision, a phenomenon that seems widespread in terminal erythropoiesis (62). Moreover, aberrant IR can be triggered by mutations to splice sites, branch points or other splicing motifs, and perturbations to splicing enhancer/repressor expression (11). However, these factors were not included in our model because we aimed to specifically determine the impact that epigenetic marks have on IR regulation.

Previous studies have mostly focused on investigating the functional links between chromatin organization and gene expression regulation, and found that NFRs at a transcription start site are strongly associated with transcription initiation (63). Nucleosomes were also reported to be preferentially positioned in exons to facilitate their identification among flanking introns by the splicing machinery (64,65). However, it is important to note that these findings were revealed using the micrococcal nuclease digestion with deep sequencing (MNase-Seq) protocol, which is more susceptible to GC content bias. Kelly *et al.* (66) showed that nucleosome enrichment in exons vs. introns was not observed in NOMe-Seq data, which they attributed to the technical differences between the two experimental approaches. NOMe-Seq data include the percentage of methylated reads at a given position as opposed to the count of mapped reads in MNase-Seq data. Similarly, our NOMe-Seq-based analysis of chromatin accessibility, quantified by GCH methylation, did not reveal a specific preference for nucleosomes to be positioned in exons rather than introns.

Our data strongly support the notion that IR is modulated through changes in chromatin accessibility. These changes could be caused by the cell type-specific action of TFs and chromatin modulators driving differentiation and polarisation of immune cells. Therefore, it should be noted that cell-intrinsic differences in chromatin accessibility might not be induced for the sole purpose of regulating IR. Our study did reveal the regions of clear GCH enrichment clusters either upstream, downstream or directly at the splice sites of retained introns in contrast to non-retained introns. High GCH methylation levels, like those observed in retained introns, are indicative of NFRs, regions of possible nucleosome eviction that are characterised by a high density of methylated GCH sites and unmethylated CpG dinucleotides (37). Interestingly, You *et al.* showed that a loss of nucleosome-depleted regions accompanied by nucleosome occupancy precedes changes in endogenous CpG methylation in *OCT4* and *NANOG* genes in the embryonic carcinoma cell line NCCIT (67). The formation of an NFR upstream from the 5′ exon/intron boundary led to DNA hypomethylation and the depletion of H3K36me3 in *SETD2*-deficient tumours (68). It is therefore reasonable to conclude that alteration of the epigenetic landscape attributed to IR initially starts with changes in nucleosome architecture and subsequent transcriptome rewiring.

Apart from signalling a nucleosome eviction, high levels of GCH methylation potentially mark regions with longer inter-nucleosomal spacing, also known as DNA linker regions. A study estimating nucleosome phasing in single cells found great agreement between average linker length measured with scNOMe-Seq and the phase estimates derived from MNase-Seq (69). Linker length ranges between ∼20 and 90 bp, varies among different species and tissues, and even fluctuates within a single cellular genome (70). Nucleosome phasing has been linked to alternative splicing before, where RNA Pol II elongation rates increase upon histone depletion, and pre-mRNA splicing is delayed (71). Previous studies identified nucleosomes as physical barriers to efficient transcription elongation *in vitro*; however, *in vivo* they are efficiently removed from transcribed chromatin (72). RNA Pol II was also found to be involved in maintaining nucleosome phasing in the transcribed region, where longer RNA Pol II dwell times, associated with slow transcription, allowed for remodelling of H3K36me3 profiles (73).

In regions further downstream of transcription start sites, nucleosome positioning becomes less stable (63) and linker region lengths become non-uniform. We therefore propose that the differences in DNA methylation and H3K36me3 signal observed in internal introns reflect the underlying changes in nucleosome organisation, that in turn propagate IR. In the presence of IR, transcription rates are faster over more spaced out nucleosomes which does not allow sufficient time for a ‘writer’ to deposit H3K36me3 in the splicing region (73). CpG sites in the DNA linker regions are usually unmethylated (69) and therefore may explain the reduced DNA methylated levels associated with IR (74).

In the proximity of transcription start sites, strong histone modification levels (like those we observed for H3K4me3 and H3K27ac) indicate a well-positioned nucleosome (75), while reduced histone modification levels, particularly reduced H3K4me3, are associated with TF binding (76). TF-binding sites can undergo nucleosome remodelling (77) in the form of nucleosome shifts or nucleosome eviction, and the formation of an NFR with associated changes to RNA Pol II elongation rates. We propose that IR in first introns might be a by-product of functional histone modifications and nucleosome remodelling for the purpose of TF recruitment in the regions proximal to transcription start sites. Interestingly, Dey and Mattick have recently identified enrichment of H3K4me3 histone modifications in short first retained introns of long non-coding RNAs (78).

In conclusion, our results advance our understanding of alternative splicing regulation. We found an unanticipated strong contribution of chromatin organization in IR modulation where nucleosomes position upstream or downstream of retained introns (determined by the length of linker regions and NFRs) to facilitate acceleration of RNA Pol II elongation and increased IR. Furthermore, the models generated in this study can be adapted to study epigenetic gene expression and alternative splicing regulation in other cell systems, other species, in health or disease, and further our understanding of these essential biological mechanisms.

## DATA AVAILABILITY

Sequencing data are deposited at the European Genome-Phenome Archive under the accession numbers EGAS00001001595 and EGAS00001001624. Access is subject to an application process as per the EGA requirements. R scripts developed for this study are available at https://github.com/combiomed/IR_code. Processed sequencing data used to train the models were deposited at Mendeley Data: http://dx.doi.org/10.17632/b6crxbxbk2.1.

## Supplementary Material

gkac994_Supplemental_Files
