# Supplementary material for: Increased chromatin accessibility facilitates intron retention in specific cell differentiation states
Source: Nucleic Acids Res. 2022 Nov 10;50(20):11563–79. doi: 10.1093/nar/gkac994 (PMC9723627; doi:10.1093/nar/gkac994)
Supplement: gkac994_Supplemental_Files [file gkac994_supplemental_files.zip › Supplementary Materials_rev2.docx]

# Supplementary Materials

**Increased chromatin accessibility facilitates intron retention in specific cell differentiation states**

Veronika Petrova^1,2^, Renhua Song^3,4^, DEEP Consortium, Karl J.V. Nordström^5^, Jörn Walter^5^, Justin J.-L. Wong^3,4^, Nicola J. Armstrong^6^, John E.J. Rasko^2,4,7^*†, Ulf Schmitz^1,2,8,9^*†

^1^ Computational BioMedicine Laboratory Centenary Institute, The University of Sydney, Camperdown 2050, Australia

^2^ Gene and Stem Cell Therapy Program Centenary Institute, The University of Sydney, Camperdown 2050, Australia

^3^ Epigenetics and RNA Biology Program Centenary Institute, The University of Sydney, Camperdown 2050, Australia

^4^ Faculty of Medicine and Health, The University of Sydney, Camperdown 2050, Australia

^5^ Laboratory of EpiGenetics, Saarland University, Campus A2 4, 66123 Saarbrücken, Germany

^6^ Mathematics and Statistics, Curtin University, Bentley, WA 6102, Australia

^7^ Cell and Molecular Therapies, Royal Prince Alfred Hospital, Camperdown 2050, Australia

^8^ Department of Molecular & Cell Biology, College of Public Health, Medical & Vet Sciences, James Cook University, Townsville 4811, Australia

^9^ Centre for Tropical Bioinformatics and Molecular Biology, Australian Institute of Tropical Health and Medicine, James Cook University, Cairns 4878, Australia

# Supplementary Tables

**Table S1 Features used for modelling IR.**

| # | Abbreviation | Feature Description |
| --- | --- | --- |
| **Intrinsic Features (mRNA-Seq)** | | |
| 1  2  3  4  5  6  7  8  9  10 | Length  Length_5p_exon  Length_3p_exon  GCcont_int  GCcont_5p_exon  GCcont_3p_exon  MaxEntScore5ss  MaxEntScore3ss  SplicedIn_5p_exon  SplicedIn_3p_exon | Intron length (continuous, bp) |
|  |  | Upstream exon length (continuous, bp) |
|  |  | Downstream exon length (continuous, bp) |
|  |  | Intron GC content (proportional, 0-1) |
|  |  | Upstream exon GC content (proportional, 0-1) |
|  |  | Downstream exon GC content (proportional, 0-1) |
|  |  | 5′ splice site strength (continuous) |
|  |  | 3′ splice site strength (continuous) |
|  |  | Upstream exon is spliced in (No – 0, Yes – 1) |
|  |  | Downstream exon is spliced in (No – 0, Yes – 1) |
| **CpG methylation Features (WGB-Seq)** | | |
| 11 | avemethint_100bp5ss | Average CpG methylation in the region +/- 100 bp from 5′ splice site (proportional, 0-1) |
| 12 | avemethint_100bp3ss | Average CpG methylation in the region +/- 100 bp from 3′ splice site (proportional, 0-1) |
| 13 | avemethint_100bpmid | Average CpG methylation in the region +/- 100 bp at the middle of an intron (proportional, 0-1) |
| 14 | CpGint_100bp5ss | Number of CpG sites in the region +/- 100 bp from 5′ splice site (count) |
| 15 | CpGint_100bp3ss | Number of CpG sites in the region +/- 100 bp from 3′ splice site (count) |
| 16 | CpGint_100bp5ss | Number of CpG sites in the region +/- 100 bp from the middle of an intron (count) |
| **Nucleosome Occupancy Features (NOMe-Seq)** | | |
| 17 | gnomePeak_olap_100bp5ss | Overlap with nucleosome peak in the region +/- 100 bp from 5′ splice site (0 – No, 1 – Yes) |
| 18 | gnomePeak_olap_100bp3ss | Overlap with nucleosome peak in the region +/- 100 bp from 3′ splice site (0 – No, 1 – Yes) |
| 19 | gnomePeak_olap_100bpmid | Overlap with nucleosome peak in the region +/- 100 bp from the middle of an intron (0 – No, 1 – Yes) |
| 20 | gnomePeak_olap_NFR_100bp5ss | Overlap with NFR in the region +/- 100 bp from 5′ splice site (0 – No, 1 – Yes) |
| 21 | gnomePeak_olap_NFR_100bp3ss | Overlap with NFR in the region +/- 100 bp from 3′ splice site (0 – No, 1 – Yes) |
| 22 | gnomePeak_olap_NFR_100bpmid | Overlap with NFR in the region +/- 100 bp from the middle of an intron (0 – No, 1 – Yes) |
| 23 | GCHmeth_100bp5ss | Average GCH methylation in the region +/- 100 bp from 5′ splice site (proportional, 0-1) |
| 24 | GCHmeth_100bp3ss | Average GCH methylation in the region +/- 100 bp from 3′ splice site (proportional, 0-1) |
| 25 | GCHmeth_100bpmid | Average GCH methylation in the region +/- 100 bp from the middle of an intron (proportional, 0-1) |
| 26 | GCHoccup_100bp5ss | Average GCH occupancy in the region +/- 100 bp from 5′ splice site (proportional, 0-1) |
| 27 | GCHoccup_100bp3ss | Average GCH occupancy in the region +/- 100 bp from 3′ splice site (proportional, 0-1) |
| 28 | GCHoccup_100bpmid | Average GCH occupancy in the region +/- 100 bp from the middle of an intron (proportional, 0-1) |

| **Histone Modification Features (ChIP-Seq)** | | | |
| --- | --- | --- | --- |
| 29 | | H3K4me1_olap_100bp5ss | Overlap with H3K4me1 signal in the region +/- 100 bp from 5′ splice site (0 – no signal, 1 – signal, 2 – strong signal) |
| 30 | | H3K4me3_olap_100bp5ss | Overlap with H3K4me3 signal in the region +/- 100 bp from 5′ splice site (0 – no signal, 1 – signal, 2 – strong signal) |
| 31 | | H3K9me3_olap_100bp5ss | Overlap with H3K9me3 signal in the region +/- 100 bp from 5′ splice site (0 – no signal, 1 – signal, 2 – strong signal) |
| 32 | | H3K27ac_olap_100bp5ss | Overlap with H3K27ac signal in the region +/- 100 bp from 5′ splice site (0 – no signal, 1 – signal, 2 – strong signal) |
| 33 | H3K27me3_olap_100bp5ss | | Overlap with H3K27me3 signal in the region +/- 100 bp from 5′ splice site (0 – no signal, 1 – signal, 2 – strong signal) |
| 34 | H3K36me3_olap_100bp5ss | | Overlap with H3K36me3 signal in the region +/- 100 bp from 3′ splice site (0 – no signal, 1 – signal, 2 – strong signal) |
| 35 | H3K4me1_olap_100bp3ss | | Overlap with H3K4me1 signal in the region +/- 100 bp from 3′ splice site (0 – no signal, 1 – signal, 2 – strong signal) |
| 36 | H3K4me3_olap_100bp3ss | | Overlap with H3K4me3 signal in the region +/- 100 bp from 3′ splice site (0 – no signal, 1 – signal, 2 – strong signal) |
| 37 | H3K9me3_olap_100bp3ss | | Overlap with H3K9me3 signal in the region +/- 100 bp from 3′ splice site (0 – no signal, 1 – signal, 2 – strong signal) |
| 38 | H3K27ac_olap_100bp3ss | | Overlap with H3K27ac signal in the region +/- 100 bp from 3′ splice site (0 – no signal, 1 – signal, 2 – strong signal) |
| 39 | H3K27me3_olap_100bp3ss | | Overlap with H3K27me3 signal in the region +/- 100 bp from 3′ splice site (0 – no signal, 1 – signal, 2 – strong signal) |
| 40 | H3K36me3_olap_100bp3ss | | Overlap with H3K36me3 signal in the region +/- 100 bp from 3′ splice site (0 – no signal, 1 – signal, 2 – strong signal) |
| 41 | H3K4me1_olap_100bpmid | | Overlap with H3K4me1 signal in the region +/- 100 bp from the middle of an intron (0 – no signal, 1 – signal, 2 – strong signal) |
| 42 | H3K4me3_olap_100bpmid | | Overlap with H3K4me3 signal in the region +/- 100 bp from the middle of an intron (0 – no signal, 1 – signal, 2 – strong signal) |
| 43 | H3K9me3_olap_100bpmid | | Overlap with H3K9me3 signal in the region +/- 100 bp from the middle of an intron (0 – no signal, 1 – signal, 2 – strong signal) |
| 44 | H3K27ac_olap_100bpmid | | Overlap with H3K27ac signal in the region +/- 100 bp from the middle of an intron (0 – no signal, 1 – signal, 2 – strong signal) |
| 45 | H3K27me3_olap_100bpmid | | Overlap with H3K27me3 signal in the region +/- 100 bp from the middle of an intron (0 – no signal, 1 – signal, 2 – strong signal) |
| 46 | H3K36me3_olap_100bp5ss | | Overlap with H3K36me3 signal in the region +/- 100 bp from 5′ splice site (0 – no signal, 1 – signal, 2 – strong signal) |

**Table S2 Experiments included in the study (separate Excel File)**

**Table S3 Epigenetic signals intersecting with 5′ splice site regions (+/- 100 bp).**

|  | | **Monoc** | | **Macro** | | **Naïve T** | | **T-CM** | | **T-EM** | |
| --- | --- | --- | --- | --- | --- | --- | --- | --- | --- | --- | --- |
|  |  | Ret | Non-Ret | Ret | Non-Ret | Ret | Non-Ret | Ret | Non-Ret | Ret | Non-Ret |
| **H3K9me3** | Strong Signal | 0 | 22 | 2 | 41 | 0 | 0 | 0 | 0 | 0 | 0 |
|  | Signal | 6 | 107 | 5 | 84 | 3 | 11 | 1 | 1 | 13 | 36 |
|  | No Signal | 1,633 | 28,267 | 812 | 30,845 | 4,816 | 20,671 | 4,944 | 18,928 | 5,634 | 18,227 |
| **H3K27me3** | Strong Signal | 7 | 21 | 2 | 11 | 0 | 0 | 0 | 0 | 0 | 0 |
|  | Signal | 9 | 108 | 5 | 92 | 0 | 2 | 1 | 4 | 0 | 1 |
|  | No Signal | 1,623 | 28,267 | 812 | 30,867 | 4,819 | 20,680 | 4,944 | 18925 | 5,647 | 18,262 |
| **H3K27ac** | Strong Signal | 46 | 596 | 23 | 1,004 | 8 | 9 | 0 | 0 | 22 | 27 |
|  | Signal | 79 | 964 | 41 | 1,094 | 50 | 201 | 36 | 66 | 150 | 291 |
|  | No Signal | 1,514 | 26,836 | 755 | 28,872 | 4,761 | 20,472 | 4,909 | 18,863 | 5,475 | 17,945 |
| **H3K36me3** | Strong Signal | 550 | 3,235 | 217 | 4,983 | 502 | 660 | 1,379 | 1,005 | 814 | 577 |
|  | Signal | 380 | 10,150 | 229 | 11,621 | 1,411 | 4,588 | 1,459 | 5,260 | 849 | 2,754 |
|  | No Signal | 709 | 15,011 | 373 | 14,366 | 2,906 | 15,434 | 2,107 | 12,664 | 3,984 | 14,932 |
| **H3K4me1** | Strong Signal | 52 | 187 | 31 | 250 | 102 | 31 | 91 | 26 | 151 | 43 |
|  | Signal | 131 | 1,009 | 87 | 1,428 | 301 | 333 | 375 | 274 | 438 | 361 |
|  | No Signal | 1,456 | 27,200 | 701 | 29,292 | 4,416 | 20,318 | 4,479 | 18,629 | 5,058 | 17,859 |
| **H3K4me3** | Strong Signal | 40 | 1,099 | 19 | 1,244 | 71 | 297 | 85 | 365 | 89 | 314 |
|  | Signal | 61 | 301 | 26 | 437 | 133 | 526 | 168 | 449 | 185 | 400 |
|  | No Signal | 1,538 | 26,996 | 774 | 29,289 | 4,615 | 19,859 | 4,692 | 18,115 | 5,373 | 17,549 |
| **NFR** | Peak | 202 | 7,841 | 90 | 7,057 | 1,502 | 5,366 | 1,528 | 4,204 | 2,052 | 5,255 |
|  | No Peak | 1,437 | 20,555 | 729 | 23,913 | 3,317 | 15,316 | 3,417 | 14,725 | 3,595 | 13,008 |

Table S4 Model performances when data is trained in one cell type and tested in another.

Elastic Net Fitted models

| Trained | **Mono** | | **Macro** | | **Naïve T** | | **T-CM** | | **T-EM** | |
| --- | --- | --- | --- | --- | --- | --- | --- | --- | --- | --- |
| Tested | AUC | Acc | AUC | Acc | AUC | Acc | AUC | Acc | AUC | Acc |
| **Mono** | 0.923 | 0.890 | 0.917 | 0.922 | 0.861 | 0.774 | 0.930 | 0.908 | 0.891 | 0.908 |
| **Macro** | 0.869 | 0.881 | 0.874 | 0.849 | 0.802 | 0.753 | 0.816 | 0.838 | 0.847 | 0.869 |
| **Naïve T** | 0.891 | 0.905 | 0.900 | 0.911 | 0.947 | 0.950 | 0.939 | 0.947 | 0.944 | 0.949 |
| **T-CM** | 0.907 | 0.913 | 0.922 | 0.923 | 0.933 | 0.868 | 0.954 | 0.958 | 0.946 | 0.949 |
| **T-EM** | 0.898 | 0.899 | 0.922 | 0.923 | 0.935 | 0.869 | 0.942 | 0.946 | 0.948 | 0.951 |

Conditional Random Forest Fitted models

|  | **Mono** | | **Macro** | | **Naïve T** | | **T-CM** | | **T-EM** | |
| --- | --- | --- | --- | --- | --- | --- | --- | --- | --- | --- |
|  | AUC | Acc | AUC | Acc | AUC | Acc | AUC | Acc | AUC | Acc |
| **Mono** | 0.942 | 0.912 | 0.907 | 0.918 | 0.647 | 0.576 | 0.895 | 0.904 | 0.894 | 0.910 |
| **Macro** | 0.870 | 0.879 | 0.905 | 0.867 | 0.787 | 0.778 | 0.816 | 0.844 | 0.842 | 0.874 |
| **Naïve T** | 0.900 | 0.903 | 0.894 | 0.901 | 0.971 | 0.946 | 0.944 | 0.945 | 0.945 | 0.951 |
| **T-CM** | 0.916 | 0.918 | 0.917 | 0.925 | 0.914 | 0.839 | 0.976 | 0.950 | 0.959 | 0.960 |
| **T-EM** | 0.916 | 0.914 | 0.921 | 0.922 | 0.939 | 0.931 | 0.943 | 0.946 | 0.975 | 0.946 |

**Table S5 Number of retained and non-retained introns per cell type under the Braunschweig(1) classification system.**

|  | Type A - Introns | | Type B - Introns | | Type C - Introns | |
| --- | --- | --- | --- | --- | --- | --- |
| **Cell Type** | Non-retained | Retained | Non-retained | Retained | Non-retained | Retained |
| Monocyte | 15,917 | 1,234 | 59 | 97 | 581 | 132 |
| Macrophage | 22,000 | 391 | 96 | 64 | 310 | 41 |
| T Naïve | 11,586 | 2,982 | 33 | 223 | 253 | 122 |
| T CM | 10,686 | 3,188 | 31 | 219 | 245 | 133 |
| T EM | 10,423 | 3,419 | 31 | 213 | 229 | 121 |

**Table S6 Experiments included in the study (separate Excel File)**

Table S7 Mean %GCH at the 5’ splice site of all and dynamically retained introns.

|  | **Intron Type** | **Monocytes** | **Macrophages** | **T Naïve** | **T Central M** | **T Effector M** |
| --- | --- | --- | --- | --- | --- | --- |
| **all** | retained | 6.97 | 16.13 | 21.12 | 19.92 | 21.34 |
|  | non-retained | 8.25 | 18.56 | 16.45 | 16.82 | 17.55 |
| **dynamic** | *Retained* | 8.34 | 17.12 | 21.07 | 21.15 | 20.80 |
|  | *Non−Retained* | 6.26 | 16.71 | 22.07 | 16.03 | 29.38 |

Table S8 Binding of lineage-determining transcription factors to intronic regions. The table shows cell type specific transcription factor binding affinities to intronic regions. Binding affinities were predicted with TEPIC (2).

|  |  | | **Affinity scores** | | | | |
| --- | --- | --- | --- | --- | --- | --- | --- |
| **Intron coordinates** | **Gene** | **TF** | **Mo** | **Ma** | **TN** | **CM** | **EM** |
| chr8: 47776983-47777685 | *PRKDC* | Pbx2:Pbx3 | 0.0077 | 0.2764 | 0.0077 | 0.0077 | 0.0077 |
| chr16: 88722066-88722217 | *PIEZO1* | Lyl1 | 0.0170 | 0.1245 | 0.0170 | 0.0170 | 0.0170 |
| chr16: 88722066-88722217 | *PIEZO1* | Snai2 | 0.0169 | 0.1238 | 0.0169 | 0.0169 | 0.0169 |
| chr11: 64258713-64258884 | *PLCB3* | Rorc:Rorb | 0.0645 | 0.0035 | 0.1238 | 0.0016 | 0.0645 |
| chr17: 1472024-1472122 | *MYO1C* | Bdp1 | 0.1142 | 0.0309 | 0.1592 | 0.0154 | 0.0839 |
| chr16: 88722066-88722217 | *PIEZO1* | Zeb1:Snai1 | 0.0149 | 0.1038 | 0.0149 | 0.0149 | 0.0149 |
| chr21: 46437081-46438163 | *PCNT* | Ascl1 | 0.0639 | 0.1006 | 0.0191 | 0.0191 | 0.0191 |
| chr10: 97400534-97401092 | *RRP12* | Rxra | 0.0549 | 0.1725 | 0.0150 | 0.0150 | 0.0150 |
| chr8: 47776983-47777685 | *PRKDC* | Sox10:Sox2 | 0.0265 | 0.1276 | 0.0004 | 0.0004 | 0.0004 |
| chr10: 97400534-97401092 | *RRP12* | Nr6a1 | 0.0247 | 0.1731 | 0 | 0 | 0 |
| chr10: 97400534-97401092 | *RRP12* | Rorc:Rorb | 0.0281 | 0.1962 | 0 | 0 | 0 |
| chr8: 47798397-47799209 | *PRKDC* | Sry | 0.1309 | 0.1281 | 0.0015 | 0.0015 | 0.0015 |
| chr8: 47798397-47799209 | *PRKDC* | Znf143:Znf76 | 0.1849 | 0.1804 | 0.0015 | 0.0015 | 0.0015 |
| chr10: 97400534-97401092 | *RRP12* | Nr1a4 | 0.0342 | 0.2329 | 0 | 0 | 0 |
| chr8: 102346395-102346902 | *UBR5* | Znf8 | 0.0121 | 0.1593 | 0.0286 | 0.0331 | 0.0121 |
| chr10: 97400534-97401092 | *RRP12* | Vdr | 0.0377 | 0.2489 | 0 | 0 | 0 |
| chr3: 49700564-49700635 | *RNF123* | Nr1d1 | 0.2332 | 0.2660 | 0.0132 | 0.0132 | 0.0132 |

Retained Introns

Non-Retained Introns

# Supplementary Figures


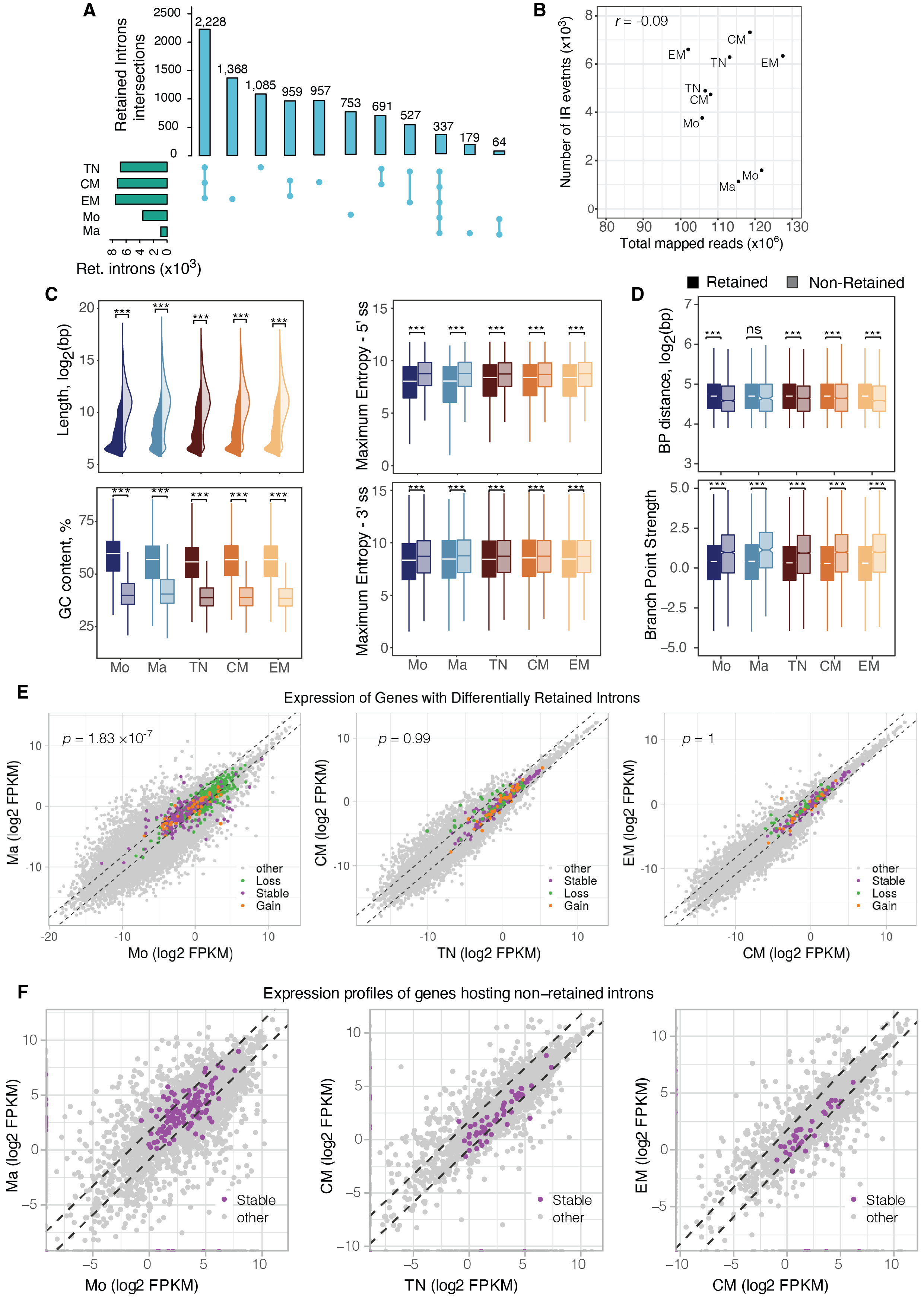


Figure S1 Characteristic of retained introns in primary immune cells. (A) UpSet plot of IR event intersections between five cell types: monocytes (Mo), macrophages (Ma), T Naïve (TN), T Central memory (CM) and T Effector Memory (EM). This plot illustrates only a subset of all possible intersections with a focus on purely myeloid or lymphoid intersections of retain introns. The bar plot on the top shows the number of IR events that are unique to one cell type or uniquely shared between multiple cell types. (B) Scatterplot of total mapped reads and the number of IR events in each sample. (C) Violin plot comparing length distributions between retained and non-retained introns and box plots of average GC content plus average 5′ and 3′ splice site strength comparison, measured by Maximum Entropy Score. (D) Branch point distance in retained vs non-retained introns and branch point strength in retained vs non-retained introns. (E) Expression profiles of the genes harbouring introns that are differentially retained during monocyte-to-macrophage differentiation or T cell maturation. Purple – host genes of introns with stable IR, green – host genes of introns with IR loss, orange – host genes of introns with IR gain. P-values are based on hypergeometric tests determining whether changes in IR events are associated with changes in gene expression. (F) Expression profiles of the genes harbouring introns that remain non-retained during monocyte-to-macrophage differentiation or T cell maturation. Purple: host genes of introns with stable non-IR. Wilcoxon signed-rank test was applied to compare lengths, GC content, and splice site strengths between retained and non-retained introns. * p<0.05, ** p<0.01, *** p<0.001. Hypergeometric test has been applied to establish associations between differential gene expression and differential intron retention.


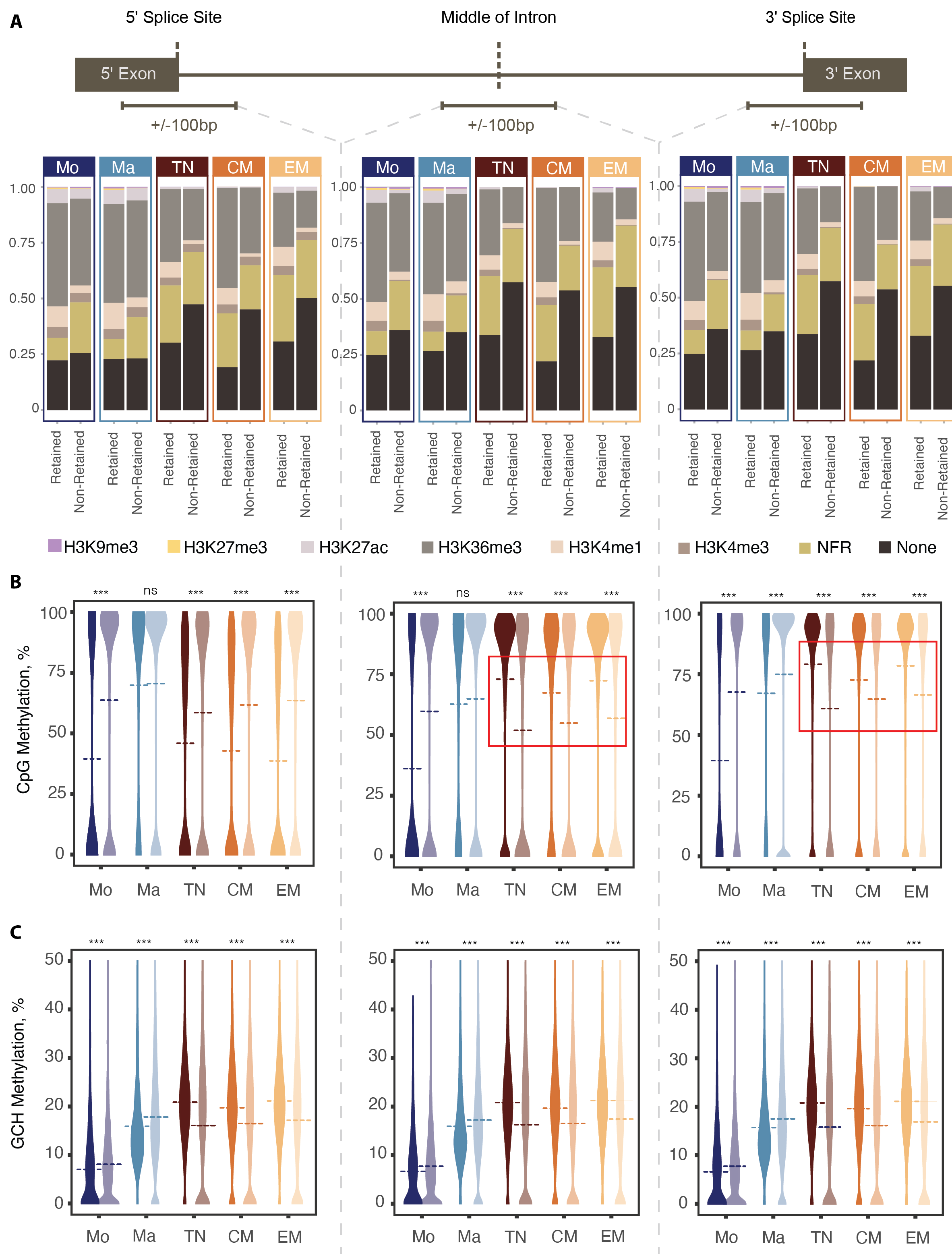


Figure S2 (A) Relative count of the HM and NFR peaks overlapping with the 5′ splice site region (left), middle (centre), and 3′ splice site region (right) of retained and non-retained introns. (B) CpG methylation distribution patterns at the 5′ splice site (left), middle (centre), and 3′ splice site (right) of retained (dark colour) and non-retained introns (light colour). Dashed horizontal lines represent mean methylation levels. Red boxes highlight opposing trends in CpG methylation in lymphoid cells. (C) GCH methylation distribution patterns at the 5′ splice site (left), middle (centre), and 3′ splice site (right) of retained and non-retained introns. Dashed horizontal lines represent mean methylation levels. Wilcoxon signed-rank test was applied to compare CpG methylation and GCH methylation between retained and non-retained introns. * p<0.05, ** p<0.01, *** p<0.001.


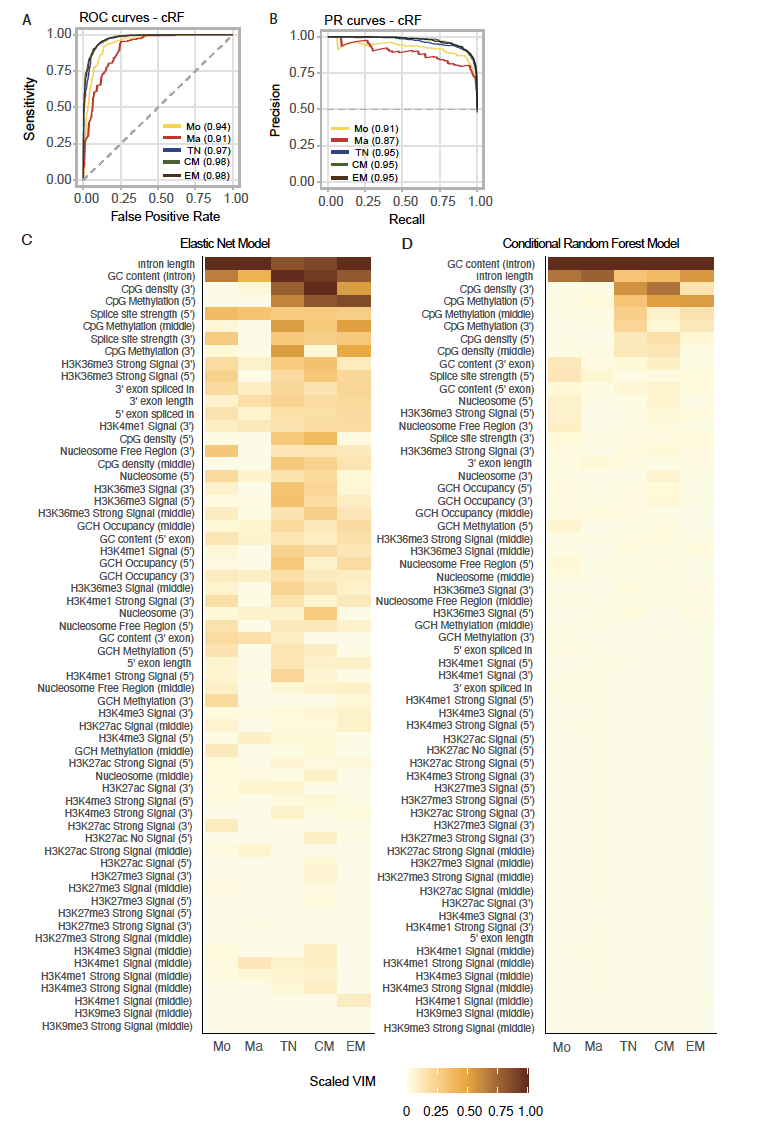


Figure S3 (A) ROC curves and (B) PR curves illustrating the performance of the cRF classifier in five cell types. Heatmap of scaled VIM values for EN models (C) and cRF models (D).


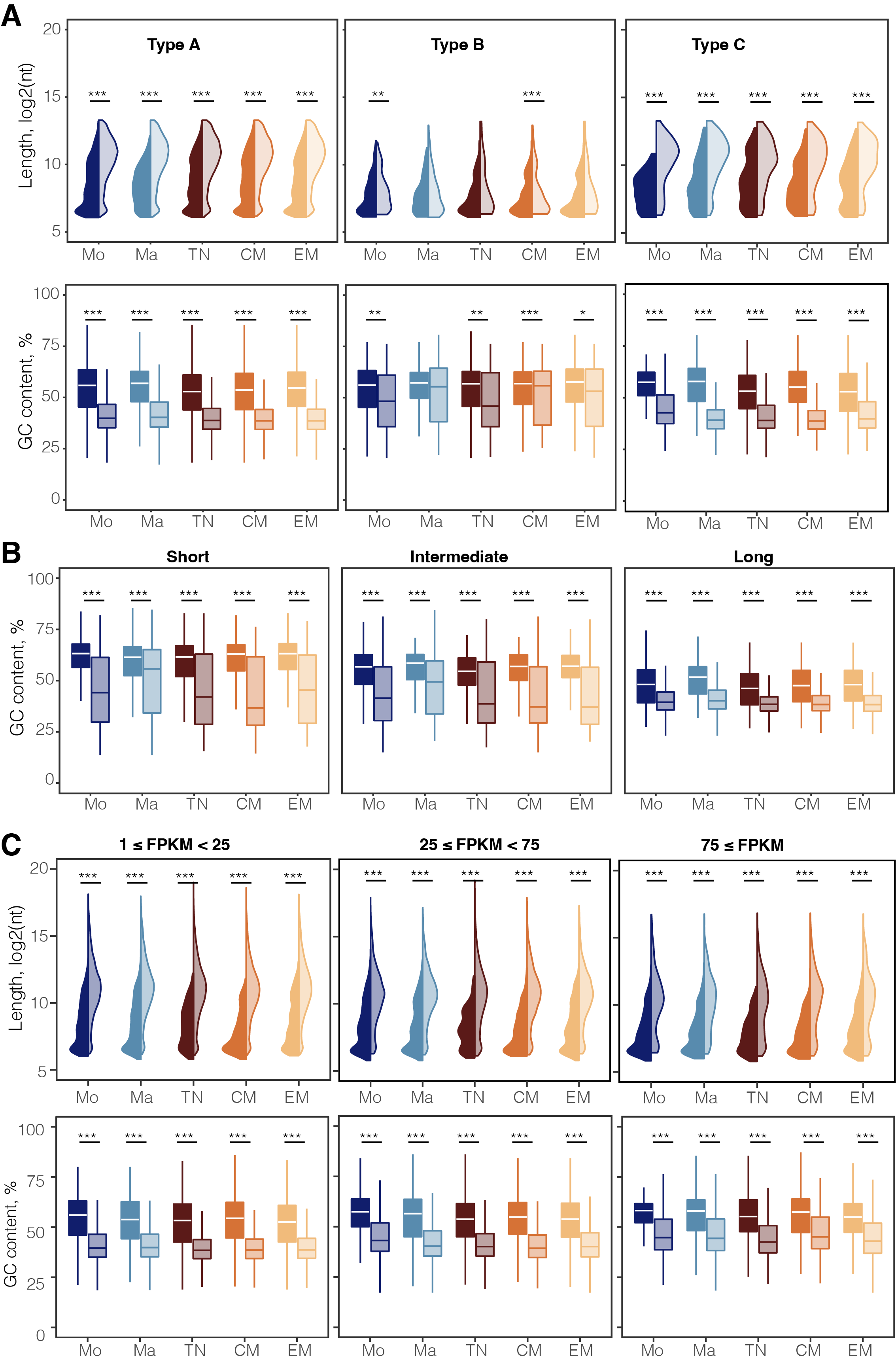


Figure S4 Intrinsic characteristics of different types of introns. (A) Length and GC content of type A, B, and C introns classified based on Braunschweig et al.(1) (dark: retained; light: non-retained). (B) Same as in (A) but for three intron length bins (short: <100nt, medium: 100-500nt, long: >500nt). (C) Same as (A) and (B) but for three host gene expression bins (lowly expressed: 1 ≤ FPKM < 25, medium 25 ≤ FPKM < 75, and highly expressed genes (FPKM ≥ 75). Wilcoxon signed-rank test was applied to compare %GC and lengths of retained and non-retained introns. * p<0.05, ** p<0.01, *** p<0.001.


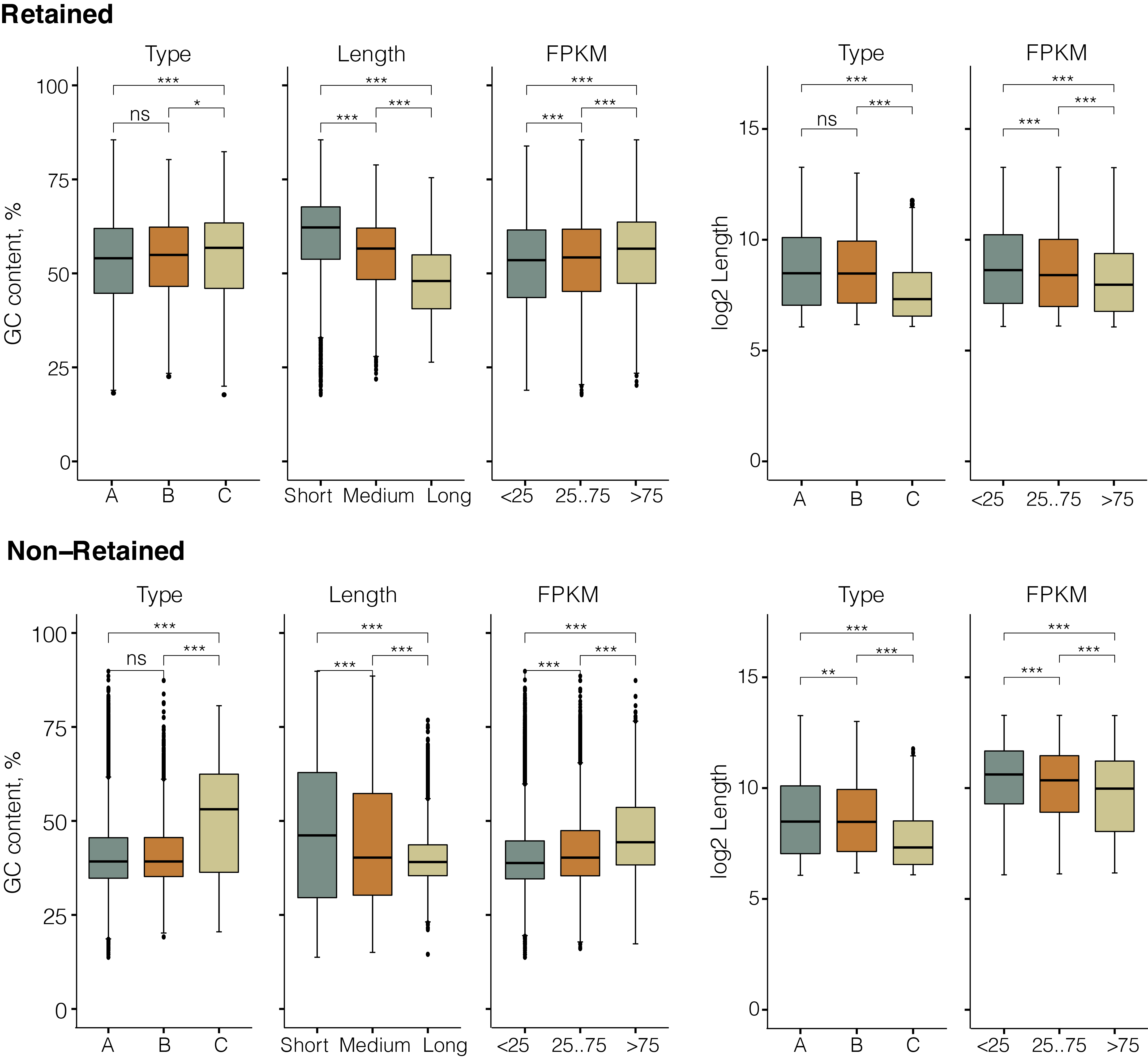


Figure S5 Comparison of GC content and length across intron types. (Top) Comparison of GC content and length in retained introns of Type A, B, and C (classified based on Braunschweig et al.(1)); short, medium, and long introns; and introns of lowly expressed, medium, and highly expressed genes. (Bottom) Comparison of GC content and length in non-retained introns of Type A, B, and C; short, medium, and long introns; and introns of lowly expressed, medium, and highly expressed genes. Wilcoxon signed-rank test was applied to compare %GC and lengths of retained and non-retained introns. * p<0.05, ** p<0.01, *** p<0.001.


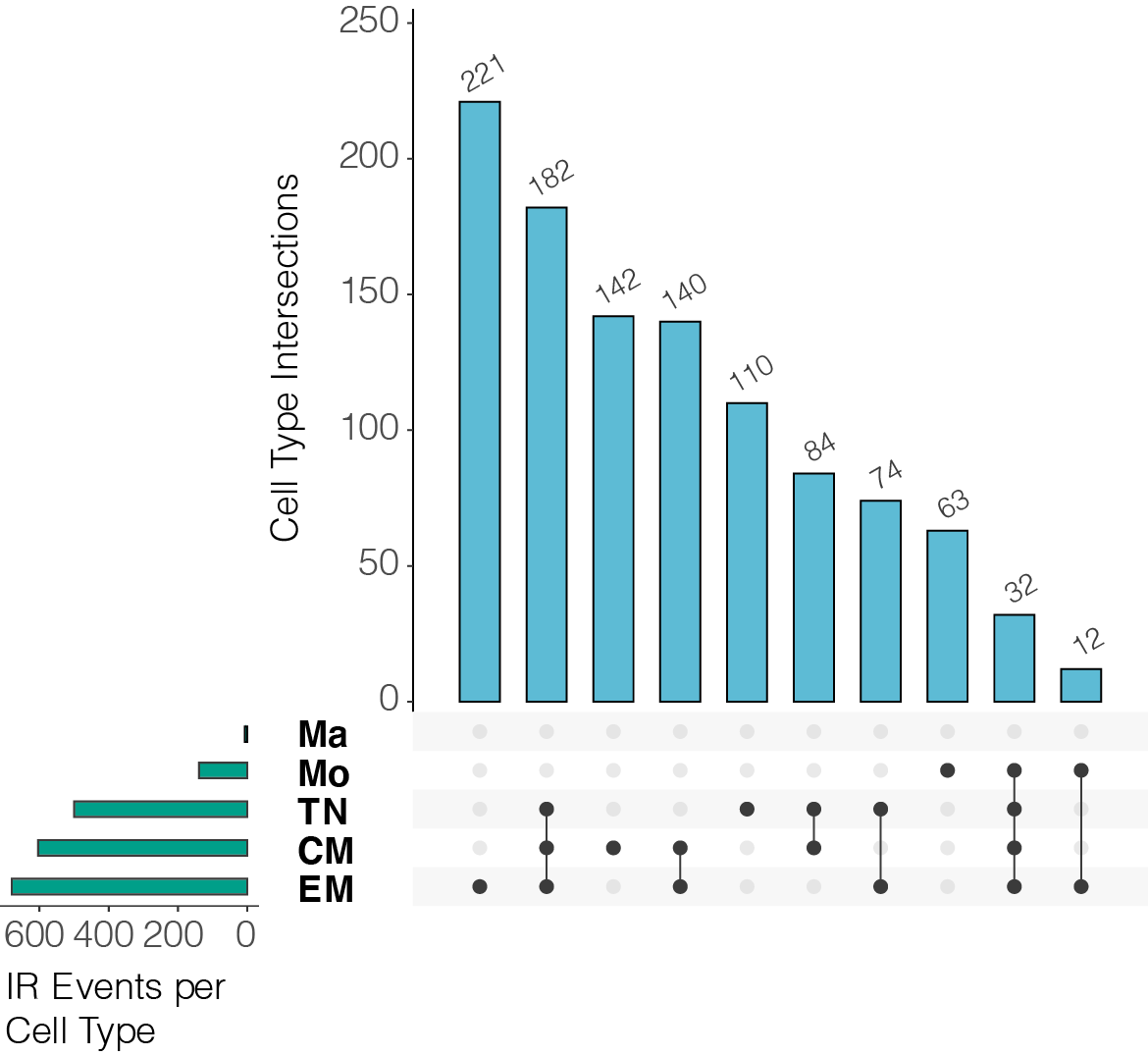


Figure S6 Upset plot of dynamic intron retention events. The bar plot on the top shows the number of IR events that are unique to one cell type or shared between multiple cell types (only the 10 largest intersections are shown).


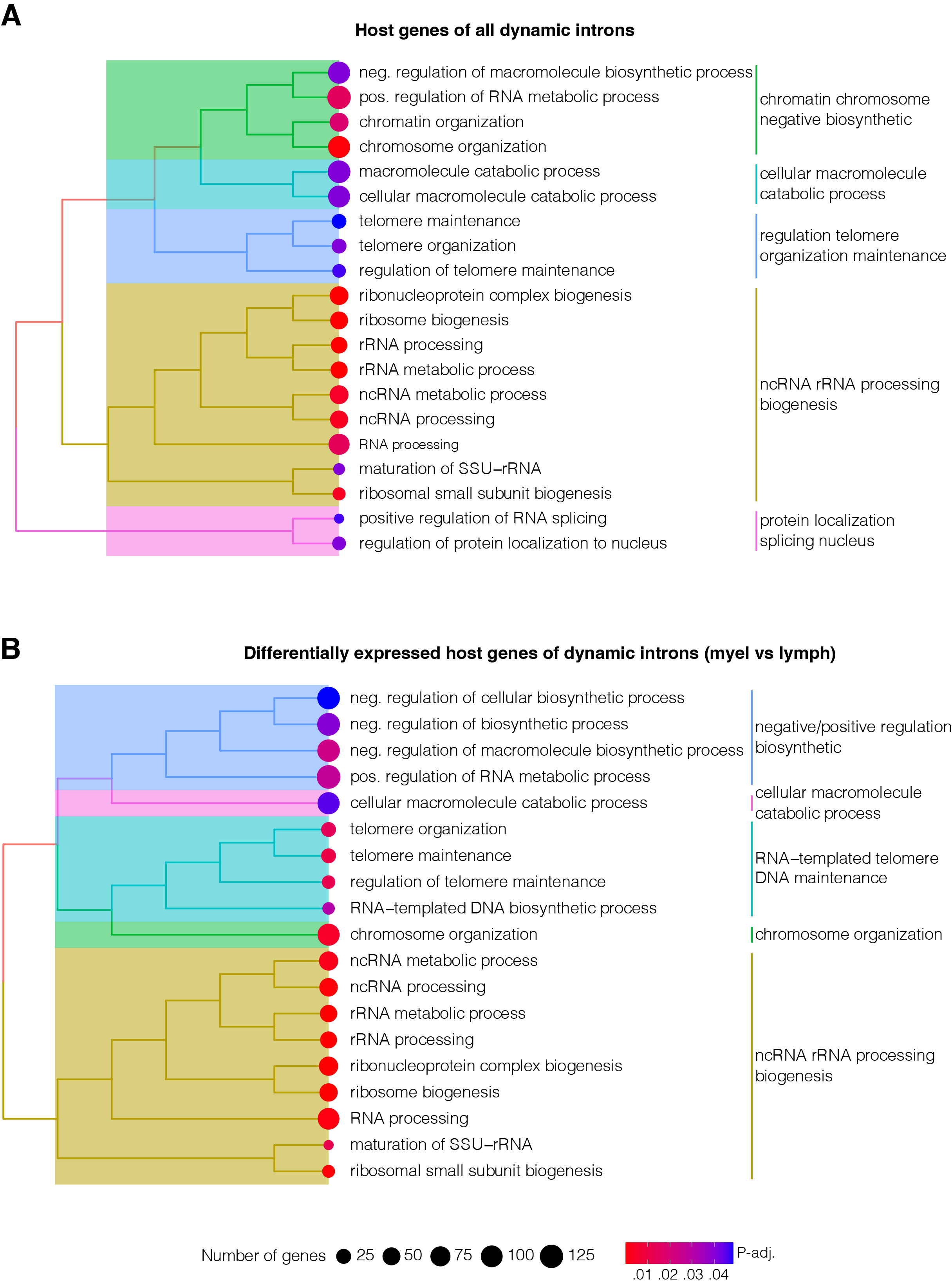


Figure S7 Gene ontology enrichment analysis of dynamic introns. (A) Enriched GO terms associated with genes hosting dynamic introns. (B) Enriched GO terms associated with genes hosting dynamic introns, that are differentially expressed between myeloid and lymphoid cells, ie. genes that are putatively affected by changing intron retention profiles.


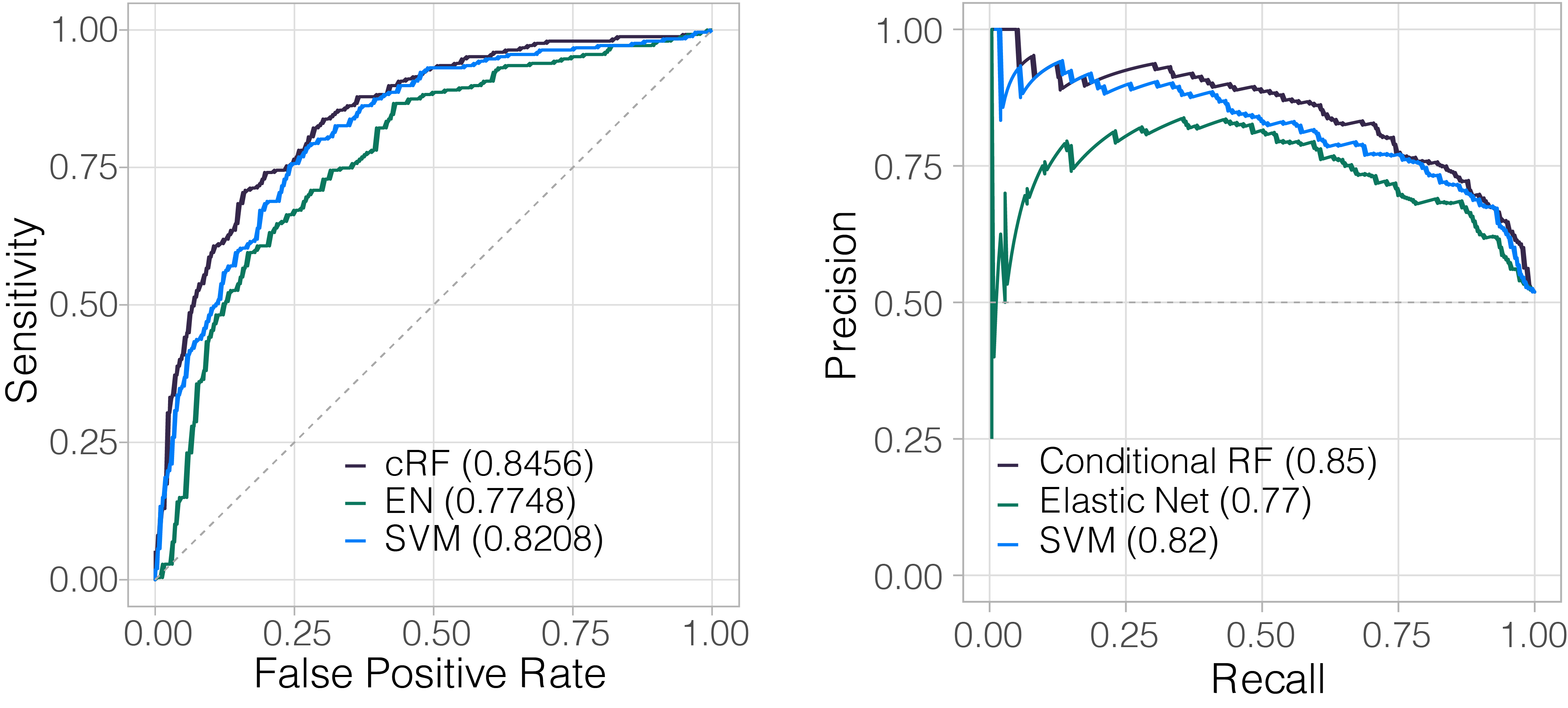


**Figure S8 Comparison of classifier performance.** Receiver operating characteristics (ROC) curves (left); precision recall (PR) curves (right).


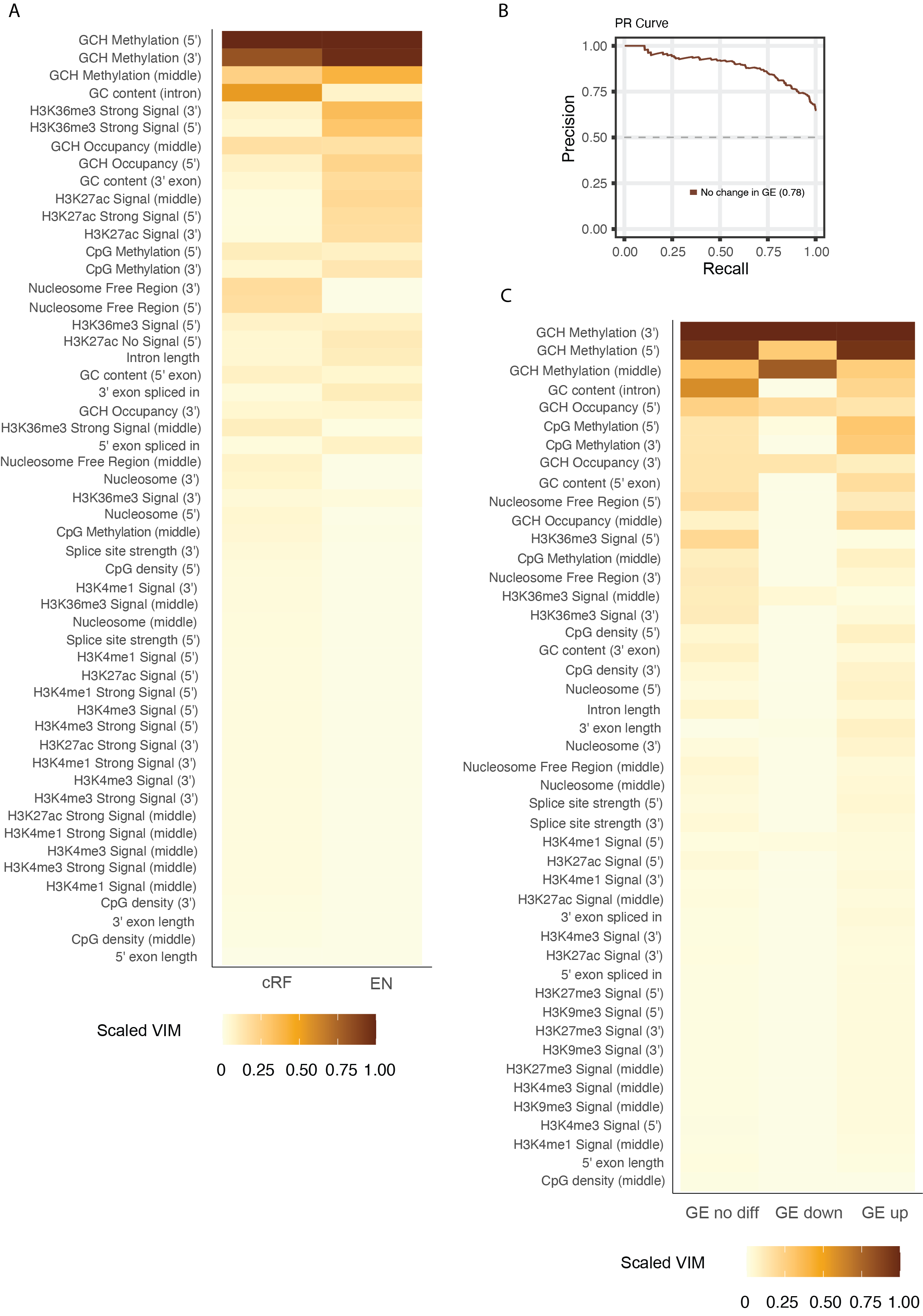


**Figure S9 (A)** Heatmap of scaled VIM values for cRF and EN models run on the dynamic introns subset. **(B)** PR curve for cRF model on dynamic introns with non-differentially expressed host genes. **(C)** Heatmap of scaled VIM values for cRF models run on three groups of dynamic introns: within non-differentially expressed genes (GE no diff), within downregulated genes (GE down), and within upregulated host genes (GE up(3)).


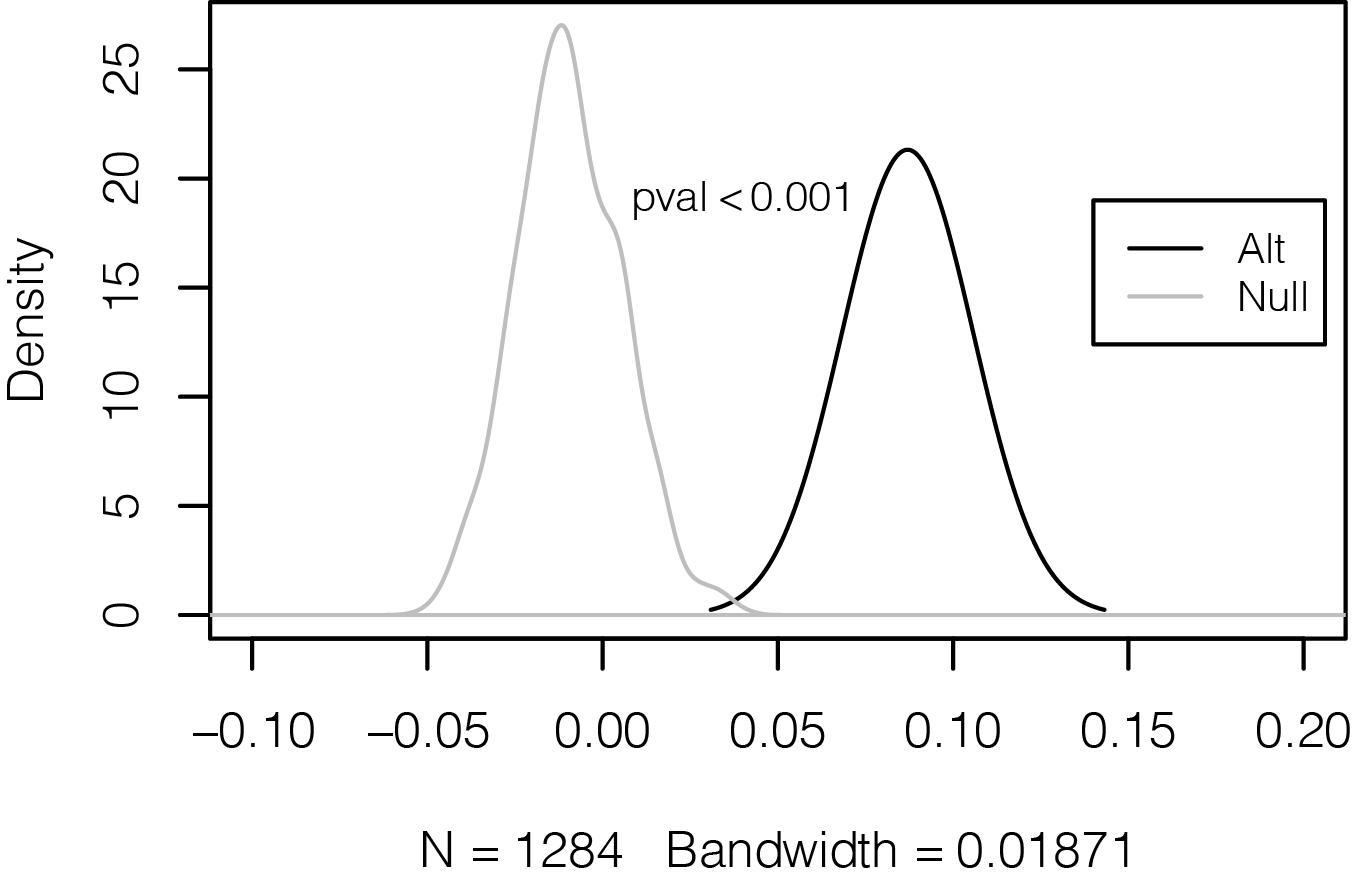


**Figure S10 Correlation between IR ratios and gene expression.** Density plot showing the distribution of correlation coefficients between IR ratios of dynamic introns and host gene expression (black curve). Correlation coefficients are significantly higher than those from a random null distribution generated from IR ratios of random introns and their host gene expression (T-test).


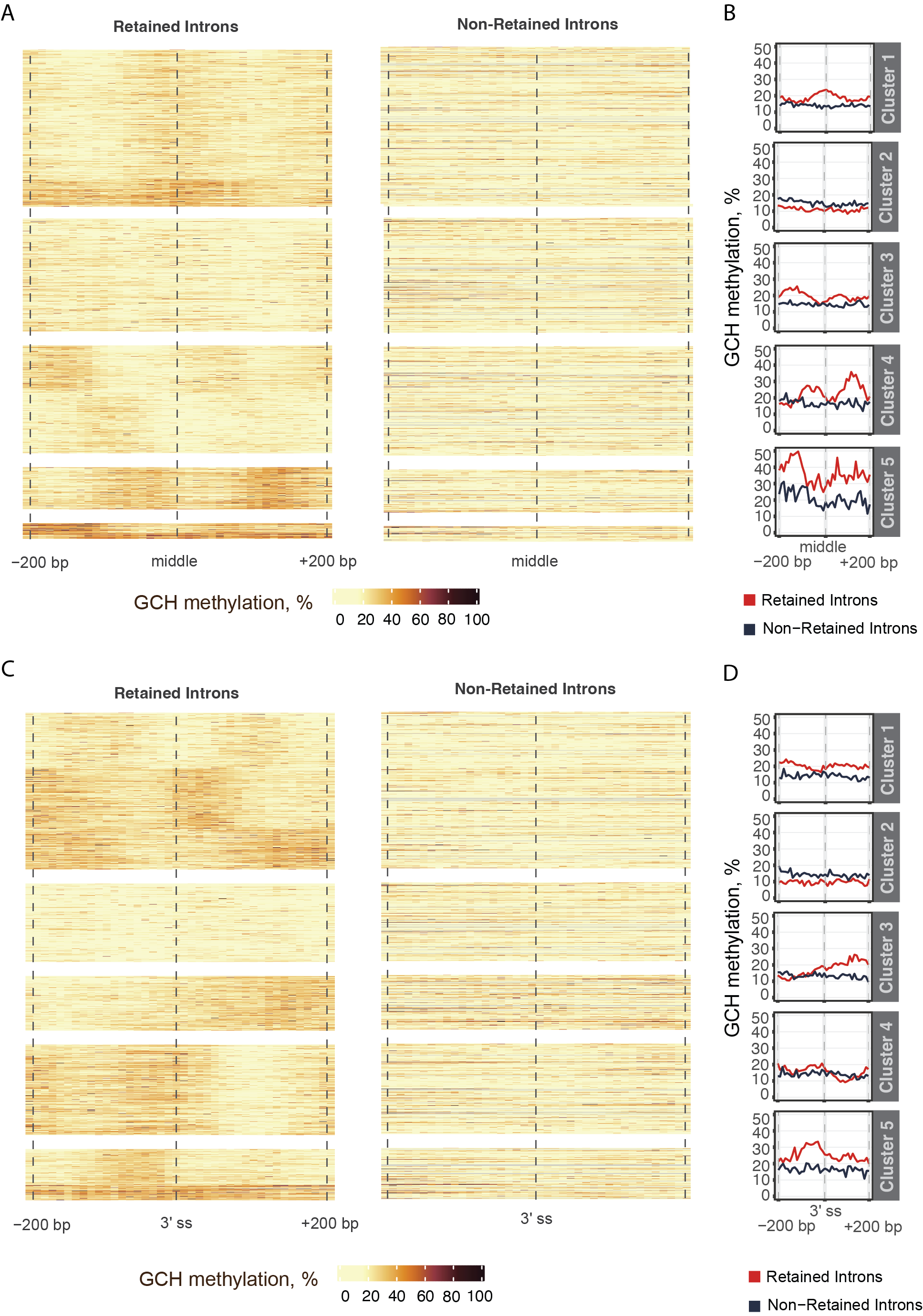


Figure S11 Clusters of GCH methylation patterns around (A) the middle (+/- 200 bp) and (C) around the 3′ ss (+/- 200 bp) of retained and non-retained introns. Line plots showing average GCH methylation values (i.e., chromatin accessibility) in retained vs non-retained introns across 5 clusters in (B) the middle and (D) at the 3′ ss.


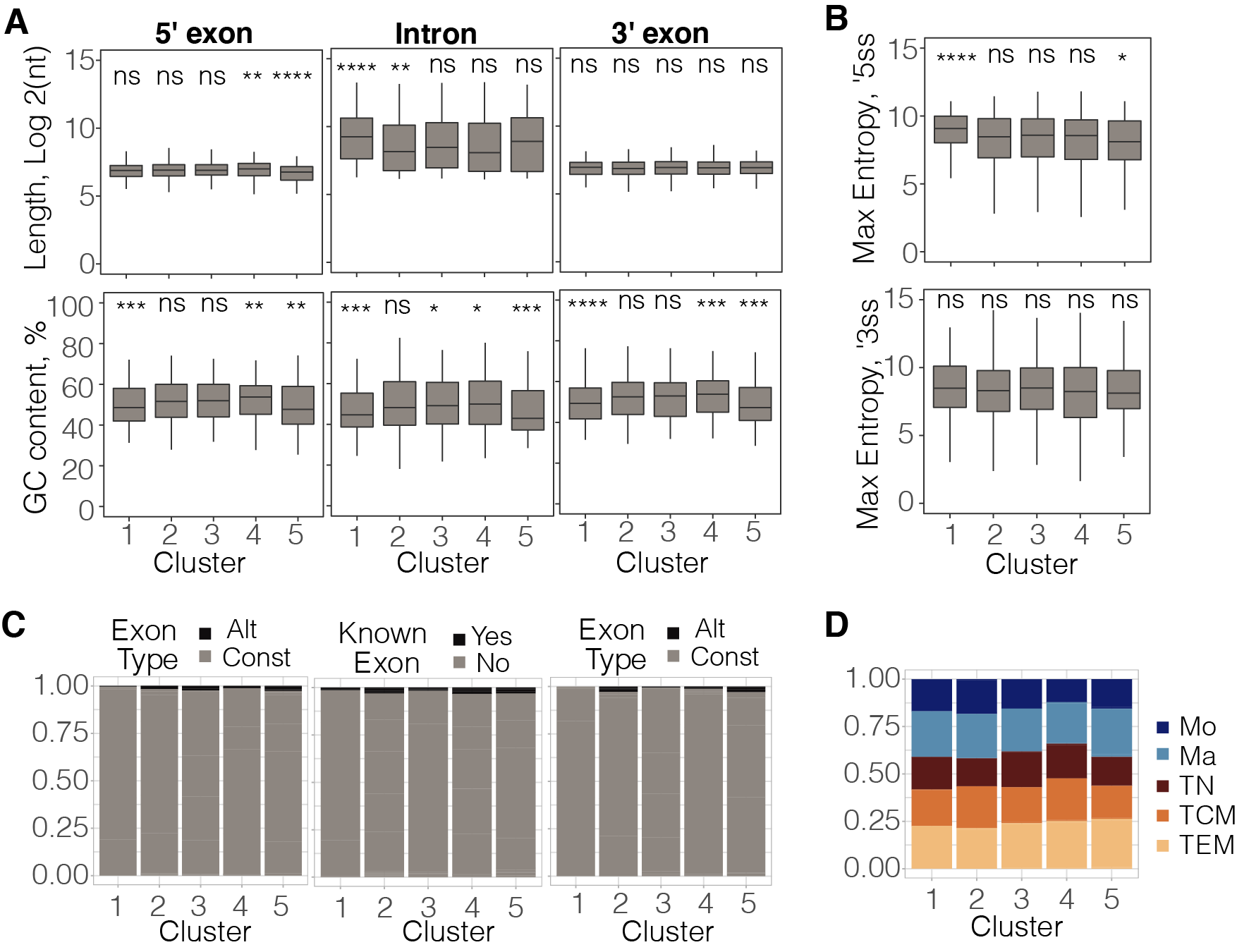


Figure S12 Features associated with GCH clusters of dynamic introns. Features analysed include length and GC content of dynamic introns and their flanking exons (A), 5ʹ and 3ʹ splice site strength (B), type of flanking exons (alternative or constitutive) and introns that overlap with known exons (in antisense transcript or alternative isoform)(C), and the cell types associated with each cluster (D).


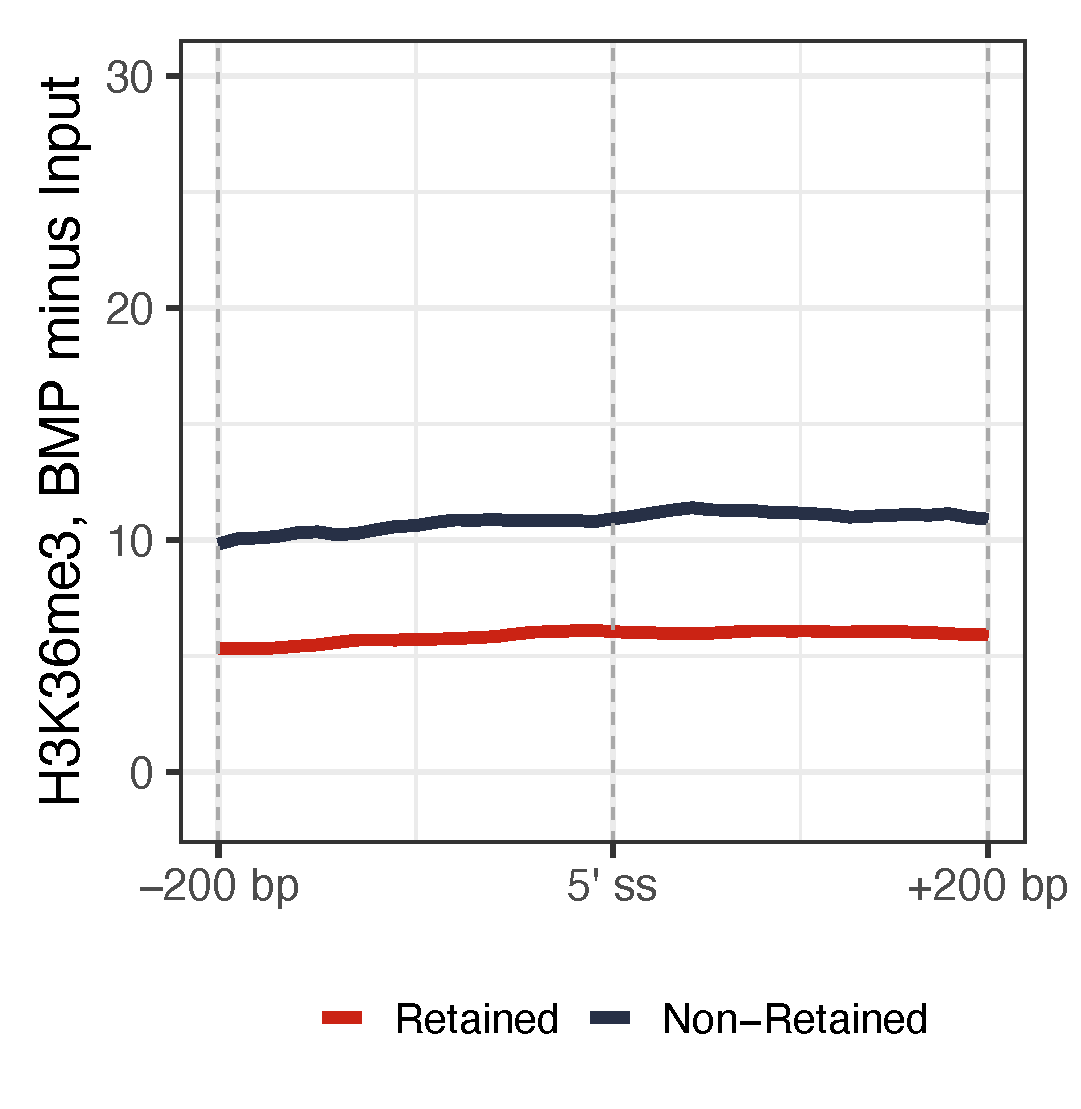

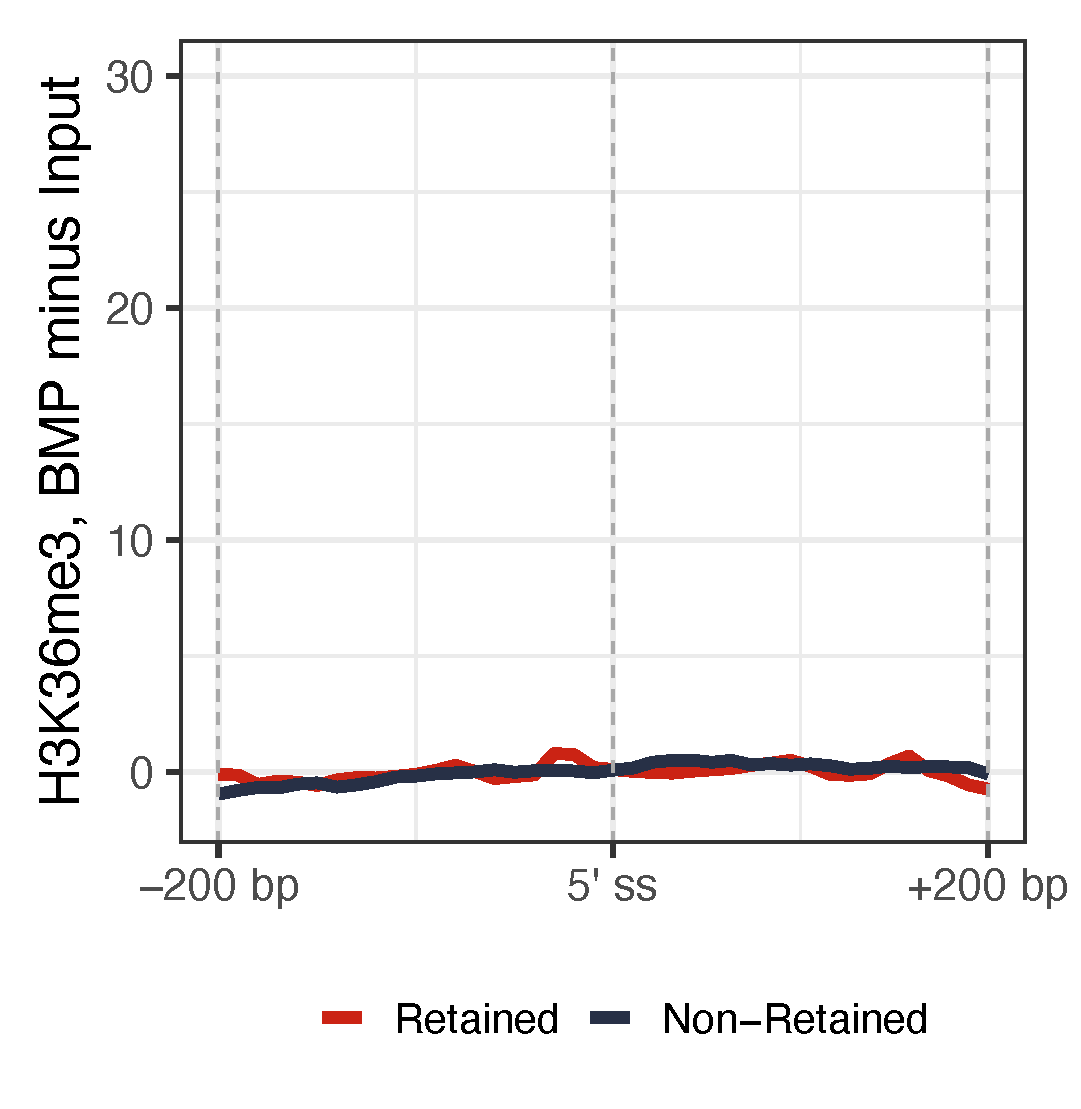


Figure S13 Normalisation based on GCH methylation suggests that H3K36me3 depends on nucleosome occupancy.

# References

1. Braunschweig, U., Barbosa-Morais, N.L., Pan, Q., Nachman, E.N., Alipanahi, B., Gonatopoulos-Pournatzis, T., Frey, B., Irimia, M. and Blencowe, B.J. (2014) Widespread intron retention in mammals functionally tunes transcriptomes. *Genome Res*, **24**, 1774-1786.

2. Schmidt, F., Kern, F., Ebert, P., Baumgarten, N. and Schulz, M.H. (2019) TEPIC 2—an extended framework for transcription factor binding prediction and integrative epigenomic analysis. *Bioinformatics*, **35**, 1608-1609.

3. Körtel, N., Rücklé, C., Zhou, Y., Busch, A., Hoch-Kraft, P., Sutandy, F.X.R., Haase, J., Pradhan, M., Musheev, M., Ostareck, D. *et al.* (2021) Deep and accurate detection of m6A RNA modifications using miCLIP2 and m6Aboost machine learning. *Nucleic Acids Research*, **49**, e92-e92.
